# Human Milk-Fed Piglets Have a Distinct Small Intestine and Circulatory Metabolome Profile Relative to That of Milk Formula-Fed Piglets

**DOI:** 10.1128/mSystems.01376-20

**Published:** 2021-02-09

**Authors:** Fernanda Rosa, Katelin S. Matazel, Ahmed A. Elolimy, Sean H. Adams, Anne Bowlin, Keith D. Williams, Lars Bode, Laxmi Yeruva

**Affiliations:** a Arkansas Children’s Nutrition Center, Little Rock, Arkansas, USA; b Department of Pediatrics, University of Arkansas for Medical Sciences, Little Rock, Arkansas, USA; c Arkansas Children’s Research Institute, Little Rock, Arkansas, USA; d Department of Microbiology and Immunology, University of Arkansas for Medical Sciences, Little Rock, Arkansas, USA; e Department of Biostatistics, University of Arkansas for Medical Sciences, Little Rock, Arkansas, USA; f Larsson-Rosenquist Foundation Mother-Milk-Infant Center of Research Excellence, University of California San Diego, La Jolla, California, USA; g Department of Pediatrics, University of California San Diego, La Jolla, California, USA; Chan Zuckerberg Biohub

**Keywords:** formula milk, gut, human milk, metabolome, serum, urine

## Abstract

The impact of human milk (HM) feeding compared with cow’s milk formula (MF) feeding on small intestinal and circulatory metabolome patterns has not been fully investigated. Therefore, 2-day-old male piglets were fed HM or MF (*n* = 26/group) from postnatal day 2 (PND 2) through 21 and were weaned to a solid diet until PND 51. The small intestine (gastrointestinal [GI]) contents, serum, and urine were collected from subsets of piglets at PND 21 and PND 51. Samples were subjected to primary metabolomics analyses at the West Coast Metabolomics Center, UC Davis. The metabolome data assessment and the statistical analyses were performed with MetaboAnalyst software. Compared with MF feeding, at PND 21, HM feeding resulted in a higher abundance of fucose in the jejunum and urine and a greater concentration of myo-inositol in serum. In HM-fed piglets, 1,5-anhydroglucitol was higher in the duodenum, serum, and urine at PND 21. Additionally, the HM group had higher levels of urinary kynurenic acid at PND 21. Correlations between bacterial genera and altered metabolites in ileum revealed that *Turicibacter* sp. and *Campylobacter* sp. were positively correlated with maltotriose and panose at PND 21, while ileal *Campylobacter* sp. was negatively correlated with fumaric acid. At PND 51, no significant metabolites were identified between HM and MF diet groups. The metabolites associated with the neonatal diets may serve as the substrates and signals that contribute to the physiological effects in HM and MF during infancy, with a subset reflecting diet-associated differences in microbial metabolism and ecology.

**IMPORTANCE** Exclusive HM feeding for newborns is recommended at least for the first 6 months of life. However, when breastfeeding is not possible, MF is recommended as a substitute. Due to the challenges associated with sample collection from infants fed HM or MF, their gut metabolism is poorly understood. Thus, an established piglet model from our team was used to determine the metabolite profile in relation to host, diet, and microbiota. The current study is the first to provide novel insights across the small intestine metabolism and its association with circulatory metabolites in the HM group relative to the MF group at the weaning and postweaning period. Data also demonstrate that during the neonatal period, diet, host, and microbial metabolism contribute to the lumen and circulatory metabolite profile. Furthermore, small intestinal lumen metabolome can be tracked in the urine as a biomarker of dietary differences, which would be a useful tool for clinical interventions.

## Abstract (precis)

Exclusive HM feeding for newborns is recommended at least for the first 6 months of life. However, when breastfeeding is not possible, MF is recommended as a substitute.

## INTRODUCTION

Human milk (HM) feeding has been associated with variety of positive health outcomes (e.g., lower incidences of allergies and enhanced immune function) in infants (1). HM contains a diversity of bioactive molecules, including lipids, human milk oligosaccharides (HMOs), a variety of cytokines (e.g., interleukin 10 and transforming growth factor β), and milk-associated microbiota that can influence the development of the immune system (2) and impact the susceptibility to infections during the neonatal stage (3). HMOs are the third most abundant component of HM after lactose and lipids and are not present in traditional formulas (4). Previous studies demonstrated that HMOs can shape the gut microbiota composition in HM-fed piglets compared with cow’s milk formula (MF)-fed animals (5, 6). Microbiota analysis of stool indicated different microbiota profiles driven by HM or MF diet in infants (7, 8). Furthermore, HMOs can be utilized by gut microbiota as prebiotics, facilitating the growth of commensal bacteria. HMOs block adhesion sites on epithelial cells or bind to surfaces of bacteria and viruses, blocking the binding of potential pathogens to the intestinal epithelium; they can also directly interact with intestinal epithelial cells, potentially enhancing intestinal development (9, 10).

The mechanisms that link HM components, the microbiome, and the infant’s development remain to be elucidated. The use of advanced molecular approaches, including metagenomics and metabolomics, could lead to novel findings about HM components and their roles in the infant’s development and overall health. In addition, several studies have used a porcine model to determine diet and microbiota interactions (6, 11–13) due to similarities in neonatal piglet and infant gastrointestinal tract development (14, 15). Furthermore, the piglet model is a valuable tool to obtain different regions of gastrointestinal tract which is limited with infants due to ethical and logistical constraints. Previous piglet studies have shown that environmental exposure has a drastic impact on gut microbiota composition (16–19) (i.e., sow-fed piglets housed at the farm compared with formula-fed piglets housed at the vivarium, sow milk, and lack of information on dietary intake). Therefore, we developed an HM-fed piglet model under controlled conditions (i.e., isocaloric diet of human milk or formula and vivarium) (2). Previous data demonstrated that HM-fed piglets had higher abundance of *Bacteroides* sp. (use HMOs) than MF-fed groups, which is similar in HM-fed infants versus MF-fed infants (6), thus supporting the use of the piglet model fed with HM. Monitoring metabolic responses to early nutrition allow us to understand the mechanisms underlying dietary intake and host-microbiota interactions. Therefore, the aim of this study was to evaluate the impact of HM feeding compared with MF feeding on small intestine and circulatory metabolome profiles of neonatal pigs.

## RESULTS

### Small intestinal metabolite profiles were altered by neonatal diet at PND 21.

**FIG 1:**
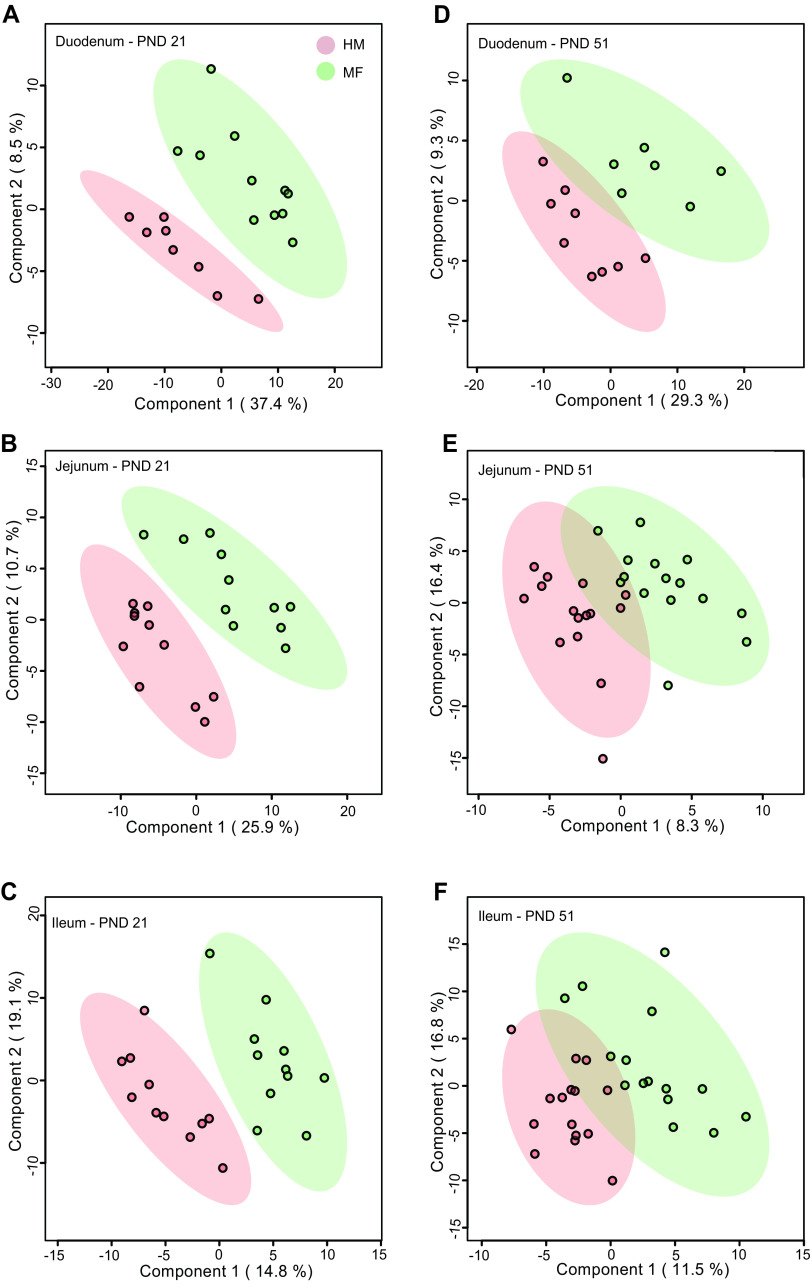
Two-dimensional score plot of partial least-squares discriminant analysis (PLS-DA) model showing how gut content abundances of known (annotated) metabolites can discriminate human milk (HM) versus milk formula (MF) feeding groups during the neonatal period in piglets. Left panels depict duodenum (A), jejunum (B), and ileum (C) contents at postnatal day 21 (PND 21). Right panels depict duodenum (D), jejunum (E), and ileum (F) at PND 51. PLS-DA scores (i.e., each symbol represents a single piglet) for PLS-DA components (dimensions) 1 and 2 are displayed. Shadows with color are 95% confidence regions. Red circles indicate individual HM piglets, and green circles indicate MF piglets. Sample numbers were *n* = 8 to 11 per group at PND 21 and *n* = 9 to 15 per group at PND 51.

To evaluate the impact of early diet on the intestinal lumen content metabolome, the duodenum, jejunum, and ileum contents were examined at PND 21 and PND 51. Supervised partial least-squares discriminant analysis (PLS-DA) using all the known (annotated) metabolites identified across the small intestinal sections revealed a clear separation between HM and MF animals at PND 21 (Fig. 1A, B, and C), indicating that variance in the levels of the gut content metabolome can discriminate diet groups from one another. The full list of metabolites significantly altered between HM and MF is presented in Table S1 in the supplemental material. At PND 21, 1,5-anhydroglucitol was greater in the duodenum of HM than the MF group. Conduritol-beta-epoxide and fucose had greater abundances in the jejunum of the HM-fed group than the MF-fed group (Table 1). Metabolites associated with bacterial metabolism, including homoserine, 4-aminobenzoic acid and benzoic acid were higher in the ileum contents of the HM- compared with the MF-fed group (Table 2). In addition, phenaceturic acid and methionine sulfoxide were higher in the ileum of the HM group than in the MF group (Table 2). The MF-fed piglets had a greater abundance of the tryptophan metabolite indole-3-propionic acid and cholesterol in the lumen of duodenum and jejunum at PND 21 (Table 2).

**TABLE 1:** Sugar metabolites significantly different between human milk or milk formula diet groups across gastrointestinal contents of piglets at postnatal day 21

Gastrointestinal content	HM^*a*^		MF^*a*^		FC^*b*^	FDR^*c*^	VIP^*d*^
	Avg abundance	SEM	Avg abundance	SEM			
Duodenum							
1,5-Anhydroglucitol	14,720	3,485	3,396	541	4.33	<0.01	2.31
Threonic acid	40,594	7,203	95,870	12,252	0.42	0.09	1.87
Maltotriose	301	69	1,416	617	0.21	0.12	1.64
Glucose	8,578	2,132	19,168	3,292	0.45	0.12	1.58
Ribonic acid	1,523	323	2,755	318	0.55	0.13	1.56
Jejunum							
Melezitose	241	41	8,326	2,680	0.03	<0.01	2.51
Fucose	231,611	94,955	20,372	3,126	11.37	0.02	2.24
Maltotriose	1,205	473	15,207	5,368	0.08	0.05	2.04
Tartaric acid	408	45	1,951	453	0.21	0.11	1.81
Panose	997	235	24,736	21,234	0.04	0.13	1.76
Conduritol-beta-expoxide	13,003	1,664	7,298	1,050	1.78	0.15	1.7
Ileum							
3,6-Anhydro-d-galactose	1,173	434	15,228	5,331	0.08	0.01	2.48
Melezitose	711	195	24,609	9,825	0.03	0.01	2.39
Maltotriose	4,535	1,796	191,435	6,8277	0.02	0.05	2.1
Panose	2,658	819	22,404	6,352	0.12	0.1	1.88
Tartaric acid	1,034	185	6,099	2,286	0.17	0.13	1.77

a Mean of normalized (mTIC) peak intensities (mz/rt) for human milk (HM) or milk formula (MF) after MetaboAnalyst analyses; *n* = 8 to 11/group.

b FC, fold change of HM mean to MF mean.

c FDR = false discovery rate; Benjamini-Hochberg-adjusted *P* value.

d VIP = variable importance in projection in PLS-DA models using all annotated metabolites to compare HM and MF within each intestinal section.

**TABLE 2:** Significantly different gastrointestinal metabolites between human milk- or milk formula-fed piglets at postnatal day 21

Gastrointestinal content	HM^*a*^		MF^*a*^		FC^*b*^	FDR^*c*^	VIP^*d*^
	Avg abundance	SEM	Avg abundance	SEM			
Duodenum							
Indole-3-propionic acid	2,520	574	4,706	386	0.54	0.09	1.81
Jejunum							
Cholesterol	1,568	321	9,917	3,480	0.16	0.11	1.85
Ileum							
Methionine sulfoxide	107,051	17,018	35,092	8,272	3.05	0.03	2.21
Cholesterol	6,240	1,372	25,956	4,881	0.24	0.04	2.14
Indole-3-propionic acid	1,379	456	3,215	850	0.43	0.1	1.88
Homoserine	2,115	291	1,101	145	1.92	0.1	1.85
Phenaceturic acid	13,348	949	8,895	1,027	1.5	0.11	1.84
Benzoic acid	120,692	36,529	25,334	5,026	4.76	0.12	1.8
4-Aminobenzoic acid	406	56	226	43	1.8	0.14	1.73

a Mean of normalized (mTIC) peak intensities (mz/rt) for human milk (HM) or milk formula (MF) after MetaboAnalyst analyses.

b Fold change of HM mean to MF mean.

c FDR, Benjamini-Hochberg-adjusted *P* value.

d VIP, variable importance in projection in PLS-DA models using all annotated metabolites to compare HM and MF within each intestinal section.

10.1128/mSystems.01376-20.5 TABLE S1 Average abundances (quantifier ion [quantion] intensities) of all metabolites significantly different when comparing human milk (HM) or milk formula (MF) diet groups, in gastrointestinal contents (duodenum, jejunum, and ileum) of piglets at postnatal day 21 (PND 21). Data are shown based on the VIP rank within each category. Download Table S1, DOCX file, 0.02 MB. Copyright © 2021 Rosa et al. 2021 Rosa et al. This content is distributed under the terms of the Creative Commons Attribution 4.0 International license.

The PLS-DA plots at PND 51 (Fig. 1D, E, and F) using annotated metabolites identified across the small intestinal sections indicated less separation between HM and MF groups in the duodenum, jejunum, and ileum, respectively. In addition, metabolites at PND 51 did not meet the false discovery rate (FDR) cutoff of ≤0.15 and variable importance in projection (VIP; >1) (see Table S2 in the supplemental material).

10.1128/mSystems.01376-20.6 TABLE S2 Average abundances (quantifier ion [quantion] intensities) of all metabolites significantly different when comparing human milk (HM) or milk formula (MF) across gastrointestinal contents (duodenum, jejunum, and ileum) of piglets at postnatal day 51 (PND 51). Data are shown based on the VIP rank within each category. Download Table S2, DOCX file, 0.02 MB. Copyright © 2021 Rosa et al. 2021 Rosa et al. This content is distributed under the terms of the Creative Commons Attribution 4.0 International license.

### Serum metabolome profile is impacted in piglets fed either human milk or dairy milk formula diet.

**FIG 2:**
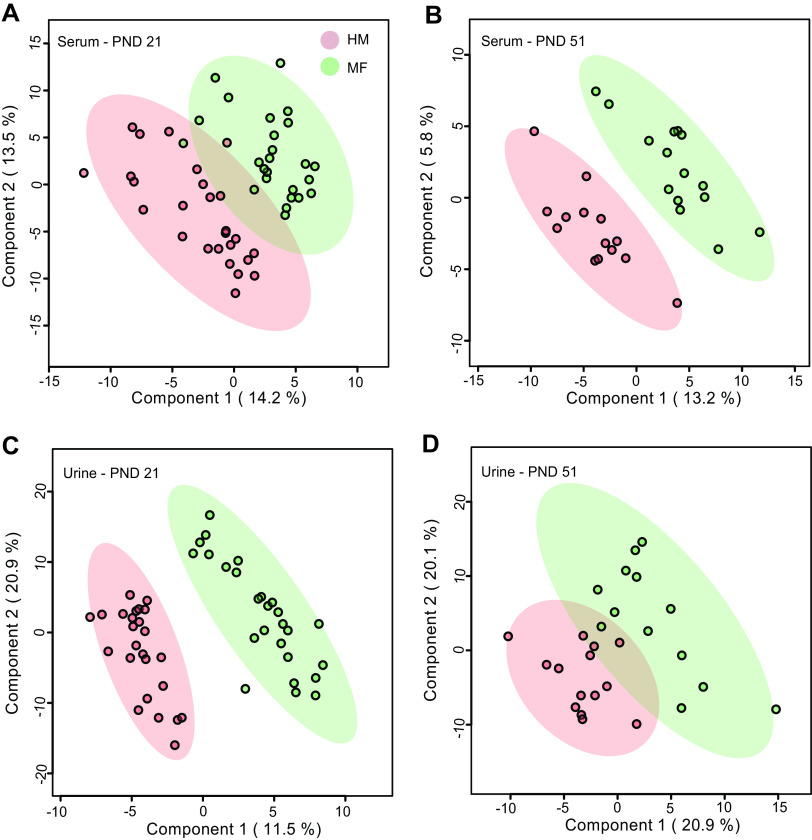
Two-dimensional score plots of partial least-squares discriminant analysis (PLS-DA) model showing that serum or urine concentrations of select metabolites can discriminate human milk (HM) and milk formula (MF) feeding groups during the neonatal period in piglets. Panels depict serum at postnatal day 21 (PND 21) (A), serum at PND 51 (B), urine at PND 21 (C), and urine at PND 51 (D). PLS-DA scores (i.e., individual piglet scores) for PLS-DA components (dimensions) 1 and 2 are displayed. Shadows with color are 95% confidence regions. Red circles indicate individual HM piglets, and green circles indicate MF piglets. Sample numbers were *n* = 25 per group at PND 21, and *n* = 15 per group at PND 51.

The PLS-DA analysis demonstrated diet-dependent separation of groups driven by variance in serum metabolites (Fig. 2A and B) (models use all annotated metabolites) profile. At PND 21, the sugar metabolites 1,5-anhydroglucitol (fold change [FC], 7.10), conduritol-beta-epoxide, myo-inositol, ribonic acid, and palmitoleic acid were higher in the serum of HM-fed piglets than in MF-fed piglets (Table 3) (FC range from 1.37 to 2.48). In contrast, fumaric and threonic acids were lower (Table 3) (FC, 0.8) in the HM group than in the MF group. At PND 51, none of the metabolites passed the false discovery rate (FDR) cutoff of ≤0.15 between diet groups (see Table S3 in the supplemental material).

**TABLE 3:** Average abundances of sugar metabolites and other metabolites significantly altered by human milk or milk formula diet in the serum of piglets^*a*^ at postnatal day 21

Metabolite by type	HM^*b*^		MF^*b*^		FC^*c*^	FDR^*d*^	VIP^*e*^
	Avg abundance	SEM	Avg abundance	SEM			
Sugar							
1,5-Anhydroglucitol	90,464	6,555	12,747	1,288	7.1	<0.01	4.76
Conduritol-beta-expoxide	2,484	200	1,141	101	2.18	<0.01	3.74
Threonic acid	40,348	1,207	50,289	1,833	0.8	<0.01	3.24
Myo-inositol	75,795	4,914	55,377	2,342	1.37	0.04	2.58
Ribonic acid	44,296	5,407	17,887	4,919	2.48	0.06	2.41
Other							
Cysteine	30,664	931	38,469	1,410	0.8	<0.01	3.27
Fumaric acid	4,559	225	5,771	332	0.79	0.06	2.49
Palmitoleic acid	1,196	144	669	99	1.79	0.06	2.42

a *n* = 25/group.

b Mean of normalized (mTIC) peak intensities (mz/rt) for human milk (HM) or milk formula (MF) after MetaboAnalyst analyses.

c Fold change of HM mean to MF mean.

d FDR, Benjamini-Hochberg-adjusted *P* value.

e VIP, variable importance in projection in PLS-DA models using all annotated metabolites to compare HM and MF within each intestinal section.

10.1128/mSystems.01376-20.7 TABLE S3 Average abundances (quantifier ion [quantion] intensities) of all serum metabolites significantly different when comparing human milk (HM) or milk formula (MF) fed piglets at postnatal day 51 (PND 51). Data are shown based on the VIP rank within each category. Download Table S3, DOCX file, 0.01 MB. Copyright © 2021 Rosa et al. 2021 Rosa et al. This content is distributed under the terms of the Creative Commons Attribution 4.0 International license.

### The urine metabolome is impacted by HM or MF infant diet.

The urine PLS-DA plot using all annotated metabolites illustrates that variance of urine metabolites can discriminate diet groups at PND 21 (Fig. 2C); this was less apparent at PND 51 (Fig. 2D). At PND 21, the abundances of monosaccharides 1,5-anhydroglucitol and fucose and the oligosaccharide raffinose were higher in urine of the HM group than that of the MF group (Table 4). The HM-fed piglets had a greater abundance of 2-hydroxyvaleric, arachidonic, glycolic, methylmalonic, gluconic, and xanthurenic acids than the MF group in urine (Table 4). Also, at PND 21, the HM neonatal diet resulted in higher levels of 2-ketoisocaproic, 3-4-hydroxyphenylpropionic acid, kynurenic acid, beta-sitosterol, and beta-tocopherol than those of the MF diet. At PND 51, with the exception of glyceric acid, which was greater in urine of HM- than that of MF-fed piglets, none of the metabolites detected were significant after FDR correction (see Table S4 in the supplemental material).

**TABLE 4:** Urinary metabolites significantly altered by diet in piglets fed with human milk or milk formula at postnatal day 21^*a*^

Urinary metabolite	HM^*b*^		MF^*b*^		FC^*c*^	FDR^*d*^	VIP^*e*^
	Avg abundance	SEM	Avg abundance	SEM			
Sugar metabolites and derivatives							
Xylonolactone	3,295	251	39,710	2,987	0.08	<0.01	3.73
Tartaric acid	8,450	1,182	180,555	13,579	0.05	<0.01	3.65
Fucose	1,884,181	283,396	356,826	35,004	5.28	<0.01	3.08
Threonic acid	252,261	23,852	513,055	31,372	0.49	<0.01	2.76
Raffinose	2,065	292	683	176	3.02	<0.01	2.74
Beta-gentiobiose	62,366	6,246	123,309	9,311	0.51	<0.01	2.7
Ribonic acid	66,882	5,118	141,502	11,306	0.47	<0.01	2.63
Glucose-1-phosphate	79,090	6,201	168,367	12,995	0.47	<0.01	2.55
2,8-Dihydroxyquinoline	5,677	977	11,039	850	0.51	<0.01	2.4
Maltotriose	2,780	538	5,954	616	0.47	<0.01	2.36
Threitol	185,405	10,665	261,302	14,255	0.71	<0.01	1.99
Erythritol	1,007,123	61,506	1,372,060	81,916	0.73	0.02	1.73
1,5-Anhydroglucitol	87,562	16,481	33,889	2,871	2.58	0.05	1.51
Sucrose	1,298	340	4,988	1,720	0.26	0.05	1.5
Gluconic acid lactone	36,145	3,782	21,400	3,110	1.69	0.05	1.48
Amino acids							
Glutamine	132,117	10,024	235,132	31,299	0.56	0.01	1.88
Phenylalanine	42,206	4,868	78,570	10,286	0.54	0.05	1.48
Homocystine	1,632	134	2,365	287	0.69	0.11	1.36
Beta-alanine	367,673	49,181	520,786	51,430	0.71	0.14	1.25
Fatty acids							
2-Hydroxyvaleric acid	370,548	36,110	199,426	24,448	1.86	<0.01	2.05
Arachidonic acid	3,969	440	2,204	250	1.8	0.01	1.87
Cis-gondoic acid	711	66	512	59	1.39	0.12	1.32
Glycerolipids							
2-Monoolein	12,692	2,480	72950	13,584	0.17	<0.01	2.66
1-Monostearin	2,054	169	3,020	331	0.68	0.02	1.66
Steroids							
Beta sitosterol	27,815	5833	2,414	386	11.52	<0.01	2.7
Cholesterol	843	88	1,710	185	0.49	<0.01	2.09
Dicarboxylic acids							
Methylmalonic acid	390,084	47,584	297,960	110,183	1.31	0.05	1.49
Adipic acid	58,881	8,810	73,217	5,214	0.8	0.13	1.3
Hydroxy acids							
Glycolic acid	111,862	12,054	70,754	5,664	1.58	0.02	1.71
2-Deoxytetronic acid	70,465	6,230	91,667	6,482	0.77	0.14	1.27
Quinoline carboxylic acids							
Xanthurenic acid	4,011	387	2,646	256	1.52	0.06	1.47
Kynurenic acid	7,616	862	5,105	548	1.49	0.14	1.25
Pyrimidines and pyridines							
6-Hydroxynicotinic acid	2,346	346	5,146	519	0.46	<0.01	2.32
4-Pyridoxic acid	1,816	881	2,384	231	0.76	<0.01	2
Cytosin	10,285	1093	6,538	681	1.57	0.05	1.48
Cytidine	2,392	341	4,653	1,121	0.51	0.14	1.28
Other metabolites							
Tocopherol beta	6,361	633	4,083	798	1.56	0.03	1.61
Urea	1,154,860	338,605	593,584	311,053	1.95	0.04	1.58
Isopropylbenzene	6,686	740	4,449	598	1.5	0.12	1.33
Triethanolamine	1,104	89	849	55	1.3	0.14	1.28
Phosphate	1,185,570	83,693	903,078	95,472	1.31	0.14	1.28
2-Ketoisocaproic acid	58,219	7,178	43,219	6,164	1.35	0.14	1.26
3-4-Hydroxyphenylpropionic acid	13,758	1,948	8,218	950	1.67	0.14	1.25

a *n* = 25/group.

b Mean of normalized (mTIC) peak intensities (mz/rt) for human milk (HM) or milk formula (MF) after MetaboAnalyst analyses.

c Fold change of HM mean to MF mean.

d FDR, Benjamini-Hochberg-adjusted *P* value.

e VIP, variable importance in projection in PLS-DA models using all annotated metabolites to compare HM and MF within each intestinal section.

10.1128/mSystems.01376-20.8 TABLE S4 Average abundances (quantifier ion [quantion] intensities) of all urinary metabolites significantly different when comparing human milk (HM) or milk formula (MF) fed piglets at postnatal day 51 (PND 51). Data are shown based on the VIP rank within each category. Download Table S4, DOCX file, 0.01 MB. Copyright © 2021 Rosa et al. 2021 Rosa et al. This content is distributed under the terms of the Creative Commons Attribution 4.0 International license.

### Correlation analysis of metabolites to microbiota at PND 21.

Our group has reported the microbiota composition in HM and MF diet groups at PND 21 from the same piglets used in the current study (6). To further explore the relationship between metabolites and gut microbiota in the small intestine, Spearman’s rho correlation analysis of diet-sensitive gut content metabolites (those identified as discriminating variables in PLS-DA models and/or significantly different using univariate statistics) and significant bacterial genera altered by diet was carried out and visualized with heat maps (color intensity and the size of the square are proportional to the correlation coefficients).

At PND 21, duodenum, jejunum, and ileum bacterial genera were significantly associated with the metabolites altered by diet in the small intestine lumen. Specifically, in the duodenum (see Fig. S1A in the supplemental material) and jejunum (Fig. S1B), the majority of detected metabolites were negatively correlated with *Campylobacter* sp. (see metabolites with green squares). Uracil was the only metabolite whose correlation with *Campylobacter* sp. in duodenum at PND 21 had an R value of ≥0.8 (see asterisk). In the ileum at PND 21 (Fig. S1C), the metabolites 3,6-anhydro-d-galactose, d-erytho-sphingosine, maltotriose, panose, melezitose, oxalic acid, and cholesterol were significantly and positively correlated with *Turicibacter* sp. Additionally, *N*-acetylglycine was negatively correlated with *Clostridium* sp. in ileum at PND 21 (Fig. S1C).

10.1128/mSystems.01376-20.1 FIG S1 Heat map showing the significant correlation values (*P ≤ *0.05) between bacterial genera and small intestine luminal metabolites in duodenum (A), jejunum (B), and ileum (C) contents at postnatal day 21 (PND 21) of piglets fed either human milk or milk formula; data from all animals were used to generate the correlation statistics (*n* = 9 to 15). Red squares represent significant positive correlations (0 < r < 1; *P ≤ *0.05), and green squares represent significant negative correlations (0 > r > −1; *P ≤ *0.05). Color intensity and the size of the square are proportional to the correlation coefficients. Only metabolites and bacteria that showed diet-associated differences in univariate and/or multivariate statistical analyses were used to generate correlations. Download FIG S1, PDF file, 0.4 MB. Copyright © 2021 Rosa et al. 2021 Rosa et al. This content is distributed under the terms of the Creative Commons Attribution 4.0 International license.

The serum metabolome altered by diet was significantly correlated with the small intestine microbiota at PND 21. Duodenum (see Fig. S2A in the supplemental material) and jejunum (Fig. S2B) *Campylobacter* sp. were correlated with diet-sensitive metabolites. In ileum (Fig. S2C), *Campylobacter*, *Clostridium*, *Turicibacter*, and *Veillonella* bacterial genera correlated (positively or negatively) with serum metabolites. Serum fumaric acid was negatively correlated with ileal *Campylobacter* sp. (Fig. S2C).

10.1128/mSystems.01376-20.2 FIG S2 Correlation heat map showing the significant correlation values (*P ≤ *0.05) between bacterial genera in duodenum (A), jejunum (B), and ileum (C) and serum metabolites at postnatal day 21 (PND 21) of piglets fed either human milk or milk formula; data from all animals were used to generate the correlation statistics (*n* = 25). Red squares represent significant positive correlations (0 < r < 1; *P ≤ *0.05), and green squares represent significant negative correlations (0 > r > −1; *P ≤ *0.05). Color intensity and the size of the square are proportional to the correlation coefficients. Only metabolites and bacteria that showed diet-associated differences in univariate and/or multivariate statistical analyses were used to generate correlations. Download FIG S2, PDF file, 0.3 MB. Copyright © 2021 Rosa et al. 2021 Rosa et al. This content is distributed under the terms of the Creative Commons Attribution 4.0 International license.

In addition, the microbiota composition identified in the lumen of the small intestine was significantly associated with the urine metabolite profile at PND 21. At PND 21, urine 1,5-anhydroglucitol was significantly and positively associated and 2-deoxytetronic acid was negatively correlated with duodenal *Campylobacter* sp. (see Fig. S3A in the supplemental material). No correlations with R value of ≥0.8 were observed between jejunum bacteria and urine metabolites (Fig. S3B). In urine, several diet-sensitive metabolites correlated with ileal *Campylobacter*, *Clostridium*, *Turicibacter*, and *Veillonella* sp. (Fig. S3C). Glutamine and homocystine in urine were positively correlated with ileal *Turicibacter* sp., and phosphate and tocopherol-beta were negatively correlated with ileal *Turicibacter* sp. (Fig. S3C). In addition, ileal *Campylobacter* sp. was negatively correlated with urine cytidine (Fig. S3C).

10.1128/mSystems.01376-20.3 FIG S3 Correlation heat map showing the significant correlation values (*P ≤ *0.05) between bacterial genera in duodenum (A), jejunum (B), and ileum (C) and urine metabolites at postnatal day 21 (PND 21) of piglets fed either human milk or milk formula; data from all animals were used to generate the correlation statistics (*n* = 25). Red squares represent significant positive correlations (0 < r < 1; *P ≤ *0.05), and green squares represent significant negative correlations (0 > r > −1; *P ≤ *0.05). Color intensity and the size of the square are proportional to the correlation coefficients. Only metabolites and bacteria that showed diet-associated differences in univariate and/or multivariate statistical analyses were used to generate correlations. Download FIG S3, PDF file, 0.5 MB. Copyright © 2021 Rosa et al. 2021 Rosa et al. This content is distributed under the terms of the Creative Commons Attribution 4.0 International license.

## DISCUSSION

Human milk shapes the neonatal microbiota, impacts gut epithelial cells by preventing pathogen adhesion (20), and influences many more aspects of metabolism and physiology in neonates. Several studies have shown that HM-fed infants have a distinct microbiota composition compared with formula-fed infants (7, 8, 21, 22). Investigating alterations in host and microbial metabolism during early life can potentially provide new insights into mechanisms behind the health outcomes in infants fed either human milk or formula. However, studies are limited in healthy infants due to gut sample collection constraints. Most recently, we have shown that formula diet likely increases small intestine inflammation, apoptosis, and tight junction disruptions and may compromise immune defense against pathogen detection in the small intestine in MF-fed piglets compared with HM-fed piglets (23). Interestingly, Chilean infants fed with cow’s milk formula versus human milk showed increased inflammatory marker expression (i.e., interleukin-8 [IL-8] and tumor necrosis factor alpha [TNF-α]) in the fecal samples (24), further supporting the data obtained in our model. These data suggest that the HM-fed piglet model shows similarities to HM-fed infants. Human and formula milk feeding can impact the metabolism (25, 26) which can be measured through metabolomics analyses in different bio-fluids that include blood (27–29), urine (30, 31), and feces (32, 33). Thus, the current study examined the circulatory, urine, and small intestine metabolite differences in piglets fed either an HM or MF diet at PND 21 and 51. A major finding was that there was a clear separation between HM and MF group on the small intestinal, serum, and urine metabolome during the exclusive milk-feeding period (PND 21) in piglets. However, at PND 51, none of the metabolites identified remained significant according to the FDR-adjusted *P* value.

In our study, HM-fed relative to MF-fed piglets had a greater abundance of the monosaccharide fucose in urine at PND 21. The mechanisms underlying the diet-associated differences in urinary fucose remain to be determined, but the urinary excretion at PND 21 might be linked to HMO metabolism. Fucose has been identified as a microbial by-product of HMOs (34, 35). Our study also indicated enrichment of the galactose pathway in serum of the HM group at PND 21. This finding may appear logical considering that metabolism of lactose, the primary sugar in breastmilk, yields glucose plus galactose. Recently, galactose has been identified as a potential HMO by-product (34), which might help explain why this sugar metabolism seems more dominant in the HM piglet group at PND 21. However, dairy milk also contains significant amounts of lactose, and the formula used in the current study included supplemental lactose and galactooligosaccharides. Interestingly, in the lower small intestine contents (jejunum and ileum), the trisaccharides melezitose and panose were more abundant in MF piglets, and throughout the small intestine, maltotriose and 3,6-anhydro-d-galactose were higher with MF. Taken together, these results and a survey of diet highlight that at PND 21, neonatal diet has a profound impact primarily on the intestinal carbohydrate milieu, which is shaped by both food sugars and microbial metabolism.

We observed a greater abundance of myo-inositol in serum of HM-fed piglets at PND 21. It is known that myo-inositol is required for the synthesis of surfactant phospholipid in immature lung tissue (36). Similarly, other studies reported a greater level of myo-inositol in the plasma of breastfed infants at 3 months of age (29) and in serum of rhesus monkeys fed human milk from birth to 3 months of age (37). Furthermore, the importance of mammalian milk-derived myo-inositol to infant growth and development has been demonstrated (38), suggesting that myo-inositol from human milk likely helps in neonatal organ development.

The greater cholesterol observed in the MF group in the small intestine (jejunum and ileum) and urine at PND 21 supports our previous finding that MF feeding induced hepatic cholesterol synthesis through the upregulation of cholesterol 7α hydroxylase (CYP7A1) protein expression in liver compared with sow-fed piglets (39). Cholesterol content in typical infant formula is lower than cholesterol in human milk (0.08 to 0.13 mmol/liter versus 0.25 to 0.46 mmol/liter) (40–43). Therefore, it is possible that the MF group increases endogenous synthesis of cholesterol in the liver to meet the requirements for growth and development compared with the HM group, as suggested previously (39). In support of this notion, several human infant studies reported that MF-fed infants had greater cholesterol synthesis than their HM-fed counterparts (43–45). Yet, the underlying mechanisms for the association between MF feeding with the greater endogenous hepatic cholesterol synthesis and greater cholesterol in the small intestine and urine in neonates needs to be investigated.

Microbial metabolites could impact gut health, metabolism, and the immune system. One example of a bioactive microbial metabolite is the tryptophan derivative indole-3-propionic acid (IPA), which is absorbed by intestinal epithelial cells and diffuses into the bloodstream, and may regulate lipid metabolism (46). In the current study, indole-3-propionic acid was higher in duodenal and ileal contents of MF at PND 21. Previously, species from *Peptostreptococcus* and *Clostridium* have been shown to convert tryptophan to IPA and appear to suppress inflammation (47–49). We have reported that genera abundance from *Peptostreptococcaceae* and *Clostridiaceae* was higher in the MF group in the small intestine at PND 21 (6). In rats fed a high-fat diet, indole-3-propionic acid supplementation blunted the high fat diet-associated intestinal epithelial barrier dysfunction, partially by decreasing the abundance of pathogenic *Bacteroides* and *Streptococcus* sp. (50). The physiological implications of these differences and the complex interactions of host and microbiota during neonatal period remain to be determined.

The gut content, blood, and urine metabolomes appeared to reflect differences in several diet components or their derivatives, as might be expected. This finding indicates that application of metabolomics has potential value in identifying biomarkers of dietary patterns. For instance, tartaric acid was lower in urine of HM-fed piglets than in MF-fed piglets, and this result was also observed in the lower small intestine contents (jejunum and ileum). Tartaric acid is not a mammalian metabolite, as approximately 85% of consumed tartaric acid is used by the intestinal microbiota, while the remaining portion is secreted in urine (30, 51). Formula is supplemented with choline bitartrate, suggesting that tartaric acid is likely from the diet at PND 21, which fits with previously published data of infants fed with MF and HM (30).

Interestingly, the greater abundance of threonic acid in MF-fed piglets in duodenum, serum, and urine at PND 21 is in agreement with results of other studies that indicated higher levels of this metabolite in the urinary metabolome of formula-fed newborns (30). High urinary levels of threonic acid have been associated with oxidative stress in adults (52), but it is important to note that threonic acid is proposed to derive from ascorbic acid metabolism (53). Since ascorbic acid was a component of the formula used here (and common to other formula recipes), higher gut and systemic levels of threonic acid with formula feeding in piglets and infants most likely reflect higher dietary ascorbate. Finally, the potential for urine metabolomics to identify markers of diet pattern is further bolstered from a survey of gut versus urine metabolites at PND 21 (i.e., comparing Tables 1 and 4). At least 17 metabolites or their derivatives (e.g., xylonolactone derivative of xylose) had the same qualitative pattern in urine and at least one segment of the small intestine. It is interesting to consider that select urinary metabolites are tracking diet-associated differences in specific regions of the gut, thus providing region-specific biomarkers and possibly a window into region-specific metabolite uptake from the gut.

In the present study, we also explored the relationship between the altered metabolites and the gut microbiota of piglets fed HM or MF. We previously reported that *Turicibacter* sp. was lower in the ileum of HM-fed piglets at PND 21 than that of the MF-fed group (6). This finding is compatible with the fact that maltotriose and panose were positively correlated with *Turicibacter* sp. at PND 21 in MF-fed piglets in the current study. In addition, an intestinal decrease in *Turicibacter* sp. was positively correlated with nutrient digestibility in sows (54). The positive correlation between duodenal *Campylobacter* sp. with serum metabolites such as ribonic acid was observed. Previously, we observed that *Campylobacter* sp. was higher in HM-fed piglet lower intestine at PND 21 (6). Regarding *Campylobacter* genera, some strains caused diarrhea in HM-fed infants (55); however, it is possible that human milk components provided a protective effect to the gut of HM-fed piglets by gut-microbiota interactions, by mechanisms yet to be determined. Future experiments that are specifically designed to determine metabolism in single microbes or defined microbe populations are required to confirm which bacteria are influencing the small intestinal and systemic metabolite milieu.

### Study limitations.

The studies were well controlled with respect to the provision of HM or MF during the neonatal period, in a manner in which body weights and growth were similar (2). Nevertheless, a solid starter pellet diet was offered to piglets in both groups from PND 14 to PND 21 along with HM or MF, which may have potentially impacted the metabolome response by a solid and liquid diet interaction. However, we anticipate that changes observed are likely related to HM or MF, as both groups received the same solid diet, and regardless of the limitation, significant diet effects on metabolite profiles were clearly observed in the gut, serum, and urine. An HM feeding model in piglets is unique and has the potential to the increase applicability of our findings to the human condition. That said, the human milk used in the current study was a pool of donor milk from mothers with 2 to 12 months of lactation and was pasteurized. It is likely that pasteurized human milk cannot mimic all of the effects of nonpasteurized HM, and this may influence metabolic or immune outcomes. However, HMOs, for example, are not degraded by pasteurization and may still have the same impact on gut physiology in pasteurized and nonpasteurized milk.

### Conclusions.

Overall, our results showed differential metabolite and food component responses to human milk or milk formula during the exclusive milk-feeding period. Future mechanistic studies are needed to determine how these metabolic changes impact early life as well as long-term health. The results also highlight the potential for urinary metabolomics to characterize biomarkers of dietary patterns during the neonatal period.

## MATERIALS AND METHODS

### Animal experiments.

The piglet study design has been previously described (2). Animal experimental procedures and treatments were conducted in accordance with the ethical guidelines for animal research approved by the Institutional Animal Care and Use Committee at the University of Arkansas for Medical Sciences. Briefly, White Dutch Landrace Duroc male piglets within 2 days old were transferred to individual housing at the Arkansas Children’s Nutrition Center vivarium. Piglets were randomly assigned to two groups (*n* = 26/diet group) of isocaloric diets composed of HM (provided from the Mother’s Milk Bank of North Texas, TX, USA) or a dairy-based MF (Similac Advance powder; Ross products, Abbott Laboratories, Columbus, OH, USA). Detailed diet composition, energy intake, and body weight gain have been previously published, and no overall difference was observed with diet intake and body weights (2). Piglets were fed 1.047 MJ/kg/day of either HM or MF every 2 h in the first week, every 4 h in the second week, and every 6 h in the third week of the study until PND 21. At PND 14, a solid food was slowly introduced (Teklad diet 140, 608; Harlan) and held steady until weaning on PND 21. Gut contents were collected at PND 21 from *n* = 11/diet group; however, serum was collected from all 26 animals/diet group. From PND 21 to PND 51, the remaining piglets were fed *ad libitum* exclusively with the same diet in accordance with the nutrient recommendations of the NRC for growing piglets (56). Tissues and samples were collected at PND 51 (*n* = 15/group). As part of a different research track determining diet effects on immune function (2), piglets were immunized on PND 21 and PND 35 with oral administration of 100 μg of cholera toxin (C8052; Millipore Sigma); 100 μg of cholera toxin subunit B (CTB; C9903; Millipore Sigma); and intramuscularly with 0.5 ml of diphtheria, tetanus, and pertussis vaccine (DTAP; Arkansas Children’s Hospital pharmacy). Within 6 to 8 h postfeeding on PND 51, piglets were euthanized (anesthetization with isoflurane, followed by exsanguination).

### Sample collection.

At PND 21 and 51, blood was collected via venipuncture of the jugular vein from anesthetized animals, into vacuum tubes. Blood samples were centrifuged at 3,000 rpm for 10 min to collect serum and stored at –80°C. Urine samples were collected by placing a small sterilized container under the cage for 2 h. The specimens were collected at room temperature, then aliquoted, and stored at –80°C until further analysis. The gastrointestinal (GI) luminal contents were separated into 3 sections, namely, duodenum, jejunum, and ileum, as previously described (6). The GI contents from each section were collected within a scintillation vial by pinching the tissue and sliding the constriction toward the open end. All samples were immediately snap frozen in liquid nitrogen and stored at –80°C until further analysis.

### Metabolomics data processing.

About 4 mg of the duodenum, jejunum, and ileum or 30 μl of serum or urine samples was extracted and subjected to untargeted metabolomics analyses using gas chromatography/mass spectrometry (GC/MS) at the West Coast Metabolomics Center at University of California Davis. Urine data were normalized to creatinine values. Detailed GC/MS instrument conditions were reported previously (57). Briefly, samples were injected into an Agilent 6890 GC equipped with a gerstel automatic liner exchange system (ALEX) that includes a multipurpose sample (MPS2) dual rail and a Gerstel CIS cold injection system (Gerstel, Muehlheim, Germany). The gas chromatograph was controlled using Leco ChromaTOF software. The gas flow rate was 1 ml/min through a 30-m-long, 0.25-mm inside diameter (i.d.) Rtx-5Sil MS column (0.25-μm 95% dimethyl 5% diphenyl polysiloxane film) with additional 10-m integrated guard column (Restek, Bellefonte PA). The transfer line temperature between gas chromatograph and mass spectrometer was set to 280°C. Electron impact was generated by a 70-eV ionization and with an ion source temperature of 250°C. Acquisition rate is 17 spectra/second, with a scan mass range of 85 to 500 Da. Following the GC data acquisition, raw peak intensities were processed in ChromaTOF versus 2.32, generating the absolute spectra intensities. The peak intensities were further assessed by a filtering algorithm in the metabolomics BinBase database using protocols previously described (58), and data were normalized by a normalization factor (mTIC), which is the sum of all peak heights for all identified metabolites (i.e., only the known compounds). For each GI section, serum, and urine, the acquired data set composed of the peak intensities was generated for a targeted mass inclusion list of metabolites with Fiehnlab BinBase database annotations (58), database identifier (i.e., InChI key [59]), the compound annotation metadata (i.e., retention index, quantification mass, BinBase identifier, and mass spectrum), and PubChem annotation (60). Quality control (QC) samples were prepared by pooling equal volumes of each sample extract for luminal contents, serum, and urine pools. Within the GI contents, serum, and urine, a total of 549 metabolites (known and unknown) were identified. A total of 282 known metabolites from diverse chemical classes, including amino acids, lipids, carbohydrates, vitamins, and cometabolites, were detected. The 267 metabolites with unknown identity were excluded from the current analysis. The complete list of the known metabolites identified at PND 21 across the intestinal regions are presented in Table S1. To check the precision of the metabolomics analyses, a supervised partial least-squares discriminant analysis (PLS-DA) was performed on the QC pools for serum, urine, and GI contents, by which each pool was composed of equal amounts of experimental samples for either serum, urine, or GI contents. The PLS-DA score plot (Fig. S4) showed a tight distribution of the QC pools during the whole experimental process.

10.1128/mSystems.01376-20.4 FIG S4 Two-dimensional score plot of partial square discriminant analysis (PLS-DA) model showing the distribution of the quality control pools used for the metabolome analysis. PLS-DA scores (i.e., individual samples) for PLS-DA components 1 and 2 are displayed. Shadows with color are 95% confidence regions. Pink circles indicate the gastrointestinal (GI) contents (including duodenum, jejunum, and ileum). Green circles indicate serum samples. Purple circles indicate urine samples. Download FIG S4, PDF file, 0.3 MB. Copyright © 2021 Rosa et al. 2021 Rosa et al. This content is distributed under the terms of the Creative Commons Attribution 4.0 International license.

### Microbiome.

Microbiota data from small intestine regions of the same animals used in the current study were published previously (6). Briefly, DNA extraction was carried out using QIAamp Fast DNA stool minikit (catalog number 51604; Germantown, MD) followed by bacterial 16S rRNA library preparation (61) and sequencing using an Illumina MiSeq instrument. The Quantitative Insight into Microbial Ecology (QIIME) pipeline was used for data analysis for operational taxonomic unit (OTU) clustering and taxonomic identification of amplicon sequences (62). We used the Greengenes 16S rRNA database, which showed a similarity threshold of 97% to cluster amplicon sequences for taxonomic annotation of OTUs.

### Statistical analysis.

The metabolome data assessment and statistical analyses were performed with MetaboAnalyst 4.0 (63). The raw data were checked for data integrity, and no missing values were detected after the peak’s filtration. Data were normalized by sum of all identified metabolites in row-wise procedures allowing adjustment for differences among samples (64), autoscaling (mean-centered and divided by standard deviation of each variable), and log transformation prior to downstream statistical analysis (65). Multivariate analysis was performed using supervised partial least-squares discriminant analysis (PLS-DA). With the PLS-DA, the difference of metabolic profiles between groups enabled the detection of group-discriminant metabolites between HM and MF treatments within each GI region contents, as well in serum and urine. The modeling included all annotated (known) metabolites. We annotated the most robust differentially abundant metabolites using the Pattern Hunter function of MetaboAnalyst using the following criteria: Benjamini-Hochberg adjusted false discovery rate (FDR) of ≤0.15 and variable importance in projection (VIP) score of >1.0 (65–68). The significant metabolites identified from the above approach were used to perform pathway enrichment analysis using MetaboAnalyst 4.0. This procedure allowed for the identification of metabolic pathways in which the differentially abundant metabolites are involved (65). On PND 51, diet and immunization interactions were assessed by permutational multivariate analysis of variance (PERMANOVA) with 999 permutations (see Table S5 in the supplemental material). Immunization did not influence the abundance of metabolites in serum, urine, and jejunum (*P* ≥ 0.3). In duodenum and ileum, we observed a diet × immunization interaction, and after Bonferroni correction, only the duodenum showed significance (*P* = 0.02). Since we did not observe an immunization effect with all samples, except for duodenal luminal contents, control and immunized animal data were pooled in the analysis of the PND 51 results. The correlations between altered metabolite abundance and bacterial genera detected in each GI region were assessed with the Spearman’s rho correlation test using the R package Corrplot. Heat maps summarizing the associations between microbial genera and metabolites at PND 21 were generated. For correlation analyses, only metabolites or bacteria that displayed diet-associated differences in terms of the criteria above were used.

10.1128/mSystems.01376-20.9 TABLE S5 Metabolites abundance in serum, urine, duodenum, jejunum, and ileum assessed by permutational multivariate ANOVA (PERMANOVA) including diet (human milk or milk formula), immunization or control (non-immunized piglets), and their interactions (diet:immunization). Download Table S5, DOCX file, 0.01 MB. Copyright © 2021 Rosa et al. 2021 Rosa et al. This content is distributed under the terms of the Creative Commons Attribution 4.0 International license.

10.1128/mSystems.01376-20.10 DATA SET S1 (Sheet 1) Relative abundance of metabolites impacted by human milk (*n* = 8) or milk formula (*n* = 11) in the duodenum contents of piglets at postnatal day 21. (Sheet 2) Relative abundance of metabolites impacted by human milk (*n* = 11) or milk formula (*n* = 11) in the jejunum contents of piglets at postnatal day 21. (Sheet 3) Relative abundance of metabolites impacted by human milk (*n* = 11) or milk formula (*n* = 11) in the ileum contents of piglets at postnatal day 21. (Sheet 4) Relative abundance of metabolites impacted by human milk (*n* = 26) or milk formula (*n* = 25) in the serum of piglets at postnatal day 21. (Sheet 5) Relative abundance of metabolites impacted by human milk (*n* = 25) or milk formula (*n* = 26) in the urine of piglets at postnatal day 21. (Sheet 6) Relative abundance of metabolites impacted by human milk (*n* = 9) or milk formula (*n* = 7) in the duodenum contents of piglets at postnatal day 51. (Sheet 7) Relative abundance of metabolites impacted by human milk (*n* = 15) or milk formula (*n* = 15) in the jejunum contents of piglets at postnatal day 51. (Sheet 8) Relative abundance of metabolites impacted by human milk (*n* = 15) or milk formula (*n* = 15) in the ileum contents of piglets at postnatal day 51. (Sheet 9) Relative abundance of metabolites impacted by human milk (*n* = 14) or milk formula (*n* = 15) in the serum of piglets at postnatal day 51. (Sheet 10) Relative abundance of metabolites impacted by human milk (*n* = 14) or milk formula (*n* = 15) in the urine of piglets at postnatal day 51. (Sheet 11) Raw peak intensities of metabolites identified by automated liner exchange and cold injection system (ALEX-CIS), gas chromatography, time of flight mass spectrometer (GCTOF MS) in human milk- or milk formula-fed piglets at postnatal days 21 and 51. Download DATA SET S1, XLSX file, 2.4 MB. Copyright © 2021 Rosa et al. 2021 Rosa et al. This content is distributed under the terms of the Creative Commons Attribution 4.0 International license.

### Data accessibility.

The raw and normalized metabolite data are available in Data Set S1 in the supplemental material.
