# Supplementary material for: Human Milk-Fed Piglets Have a Distinct Small Intestine and Circulatory Metabolome Profile Relative to That of Milk Formula-Fed Piglets
Source: mSystems. 2021 Feb 9;6(1):e01376-20. doi: 10.1128/mSystems.01376-20 (PMC7883546; doi:10.1128/mSystems.01376-20)
Supplement: TABLE S1 [file mSystems.01376-20-st001.docx]

| **Duodenum** | **HM^1^** | **SEM^2^** | **MF^1^** | **SEM^2^** | **FC^3^** | **FDR^4^** | **VIP^5^** |
| --- | --- | --- | --- | --- | --- | --- | --- |
| 1,5-anhydroglucitol | 14720 | 3485 | 3396 | 541 | 4.33 | < 0.01 | 2.31 |
| adenosine | 276 | 37 | 508 | 38 | 0.54 | 0.09 | 1.93 |
| erythronic acid lactone | 2228 | 456 | 5090 | 675 | 0.44 | 0.09 | 1.89 |
| threonic acid | 40594 | 7203 | 95870 | 12252 | 0.42 | 0.09 | 1.87 |
| maltitol | 219 | 46 | 1243 | 621 | 0.18 | 0.09 | 1.82 |
| indole-3-propionic acid | 2520 | 574 | 4706 | 386 | 0.54 | 0.09 | 1.81 |
| uracil | 162512 | 20046 | 286901 | 24588 | 0.57 | 0.09 | 1.80 |
| melibiose | 136 | 30 | 517 | 204 | 0.26 | 0.12 | 1.73 |
| 3-hydroxybenzoic acid | 260 | 53 | 523 | 67 | 0.50 | 0.12 | 1.71 |
| sorbitol | 9481 | 1507 | 33891 | 9046 | 0.28 | 0.12 | 1.69 |
| 2-monoolein | 78819 | 56783 | 199185 | 59003 | 0.40 | 0.12 | 1.69 |
| tocopherol-gamma | 477 | 69 | 2075 | 551 | 0.23 | 0.12 | 1.67 |
| serotonin | 1277 | 391 | 3367 | 598 | 0.38 | 0.12 | 1.66 |
| pyruvic acid | 5012 | 639 | 9518 | 1366 | 0.53 | 0.12 | 1.65 |
| digalacturonic acid | 304 | 56 | 670 | 98 | 0.45 | 0.12 | 1.65 |
| maltotriose | 301 | 69 | 1416 | 617 | 0.21 | 0.12 | 1.64 |
| gluconic acid lactone | 453 | 103 | 732 | 75 | 0.62 | 0.12 | 1.62 |
| uridine | 13660 | 6398 | 35372 | 8733 | 0.39 | 0.12 | 1.62 |
| deoxycholic acid | 312 | 60 | 753 | 164 | 0.41 | 0.12 | 1.62 |
| 2-picolinic acid | 663 | 202 | 2669 | 608 | 0.25 | 0.12 | 1.60 |
| beta sitosterol | 7815 | 3906 | 31733 | 9770 | 0.25 | 0.12 | 1.60 |
| glucose | 8578 | 2132 | 19168 | 3292 | 0.45 | 0.12 | 1.58 |
| UDP-glucuronic acid | 20875 | 6974 | 126049 | 49460 | 0.17 | 0.13 | 1.57 |
| ribonic acid | 1523 | 323 | 2755 | 318 | 0.55 | 0.13 | 1.56 |
| xylose | 5139 | 805 | 11188 | 2681 | 0.46 | 0.13 | 1.56 |
| lactobionic acid | 1915 | 478 | 5729 | 1147 | 0.33 | 0.14 | 1.53 |
| 3,6-anhydro-D-galactose | 786 | 222 | 3908 | 1445 | 0.20 | 0.14 | 1.53 |
| arachidic acid | 16813 | 2894 | 25919 | 2551 | 0.65 | 0.15 | 1.51 |
| **Jejunum** |  |  |  |  |  |  |  |
| 3,6-anhydro-D-galactose | 605 | 101 | 3790 | 744 | 0.16 | < 0.01 | 2.61 |
| melezitose | 241 | 41 | 8326 | 2680 | 0.03 | < 0.01 | 2.51 |
| uracil | 111134 | 9322 | 241490 | 32031 | 0.46 | 0.02 | 2.32 |
| tocopherol-gamma | 1224 | 267 | 9960 | 2379 | 0.12 | 0.02 | 2.31 |
| pinitol | 441 | 50 | 1222 | 195 | 0.36 | 0.02 | 2.28 |
| fucose | 231611 | 94955 | 20372 | 3126 | 11.37 | 0.02 | 2.24 |
| shikimic acid | 3966 | 476 | 10236 | 1812 | 0.39 | 0.03 | 2.18 |
| orotic acid | 1039 | 250 | 11622 | 6548 | 0.09 | 0.03 | 2.13 |
| maltotriose | 1205 | 473 | 15207 | 5368 | 0.08 | 0.05 | 2.04 |
| homocystine | 380 | 23 | 678 | 94 | 0.56 | 0.05 | 2.03 |
| aminomalonate | 43783 | 2985 | 60742 | 3307 | 0.72 | 0.05 | 2.02 |
| 2,5-dihydroxypyrazine | 2159 | 104 | 3105 | 294 | 0.70 | 0.11 | 1.89 |
| deoxycholic acid | 989 | 190 | 2542 | 544 | 0.39 | 0.11 | 1.87 |
| cholesterol | 1568 | 321 | 9917 | 3480 | 0.16 | 0.11 | 1.85 |
| galactinol | 4266 | 744 | 31419 | 21634 | 0.14 | 0.11 | 1.83 |
| erythritol | 4415 | 429 | 9020 | 1363 | 0.49 | 0.11 | 1.82 |
| tartaric acid | 408 | 45 | 1951 | 453 | 0.21 | 0.11 | 1.81 |
| threitol | 1227 | 127 | 2057 | 267 | 0.60 | 0.11 | 1.81 |
| 1-methyladenosine | 255 | 43 | 407 | 41 | 0.63 | 0.13 | 1.77 |
| panose | 997 | 235 | 24736 | 21234 | 0.04 | 0.13 | 1.76 |
| 2-ketoisocaproic acid | 12594 | 470 | 15627 | 922 | 0.81 | 0.13 | 1.75 |
| 2,6-diaminopimelic acid | 342 | 33 | 602 | 92 | 0.57 | 0.14 | 1.72 |
| conduritol-beta-expoxide | 13003 | 1664 | 7298 | 1050 | 1.78 | 0.15 | 1.70 |
| mucic acid | 99 | 14 | 284 | 130 | 0.35 | 0.15 | 1.69 |
| oleamide | 885 | 94 | 1632 | 329 | 0.54 | 0.15 | 1.69 |
| **Ileum** |  |  |  |  |  |  |  |
| 3,6-anhydro-D-galactose | 1173 | 434 | 15228 | 5331 | 0.08 | 0.01 | 2.48 |
| isopropylbenzene | 24144 | 5189 | 5347 | 590 | 4.52 | 0.01 | 2.41 |
| melezitose | 711 | 195 | 24609 | 9825 | 0.03 | 0.01 | 2.39 |
| acetophenone | 28432 | 6574 | 4501 | 529 | 6.32 | 0.01 | 2.34 |
| N-acetylglycine | 5633 | 373 | 2303 | 458 | 2.45 | 0.03 | 2.22 |
| methionine sulfoxide | 107051 | 17018 | 35092 | 8272 | 3.05 | 0.03 | 2.21 |
| cholesterol | 6240 | 1372 | 25956 | 4881 | 0.24 | 0.04 | 2.14 |
| maltotriose | 4535 | 1796 | 191435 | 68277 | 0.02 | 0.05 | 2.10 |
| monomyristin | 2068 | 391 | 672 | 102 | 3.08 | 0.06 | 2.06 |
| butylamine | 8227 | 1639 | 3350 | 337 | 2.46 | 0.07 | 2.01 |
| palmitoleic acid | 1526 | 282 | 655 | 120 | 2.33 | 0.07 | 2.00 |
| D-erythro-sphingosine | 16710 | 3434 | 48324 | 9314 | 0.35 | 0.09 | 1.93 |
| methylmalonic acid | 241494 | 15463 | 168841 | 15943 | 1.43 | 0.09 | 1.92 |
| panose | 2658 | 819 | 22404 | 6352 | 0.12 | 0.10 | 1.88 |
| indole-3-propionic acid | 1379 | 456 | 3215 | 850 | 0.43 | 0.10 | 1.88 |
| cytosine | 7019 | 2213 | 2312 | 353 | 3.04 | 0.10 | 1.86 |
| homoserine | 2115 | 291 | 1101 | 145 | 1.92 | 0.10 | 1.85 |
| phenaceturic acid | 13348 | 949 | 8895 | 1027 | 1.50 | 0.11 | 1.84 |
| 6-hydroxynicotinic acid | 429 | 35 | 283 | 42 | 1.51 | 0.12 | 1.80 |
| benzoic acid | 120692 | 36529 | 25334 | 5026 | 4.76 | 0.12 | 1.80 |
| tartaric acid | 1034 | 185 | 6099 | 2286 | 0.17 | 0.13 | 1.77 |
| 2,8-dihydroxyquinoline | 1163 | 337 | 6558 | 1755 | 0.18 | 0.13 | 1.76 |
| oxalic acid | 15809 | 2635 | 37082 | 7448 | 0.43 | 0.13 | 1.75 |
| 4-aminobenzoic acid | 406 | 56 | 226 | 43 | 1.80 | 0.14 | 1.73 |

^1^Mean of normalized (mTIC) peak intensities (mz/rt) for human milk (HM) or milk formula (MF) after MetaboAnalyst analyses; n=8-11/group

^2^SEM = Standard error of the mean

^3^Fold change of HM mean to MF mean

^4^FDR = Benjamini-Hochberg adjusted P-Value

^5^VIP = variable importance in projection in PLS-DA models using all annotated metabolites to compare HM and MF within each bio-region. The table only presents metabolites with significant differences between diet groups; all detected metabolites are provided in Supplemental dataset S1.
