# Supplementary material for: Human Milk-Fed Piglets Have a Distinct Small Intestine and Circulatory Metabolome Profile Relative to That of Milk Formula-Fed Piglets
Source: mSystems. 2021 Feb 9;6(1):e01376-20. doi: 10.1128/mSystems.01376-20 (PMC7883546; doi:10.1128/mSystems.01376-20)
Supplement: TABLE S2 [file mSystems.01376-20-st002.docx]

| **Duodenum** | **HM^1^** | **SEM^2^** | **MF^1^** | **SEM^2^** | **FC^3^** | **FDR^5^** | **VIP^6^** |
| --- | --- | --- | --- | --- | --- | --- | --- |
| dehydroascorbic acid | 28013 | 4371 | 6150 | 1763 | 4.56 | 0.27 | 2.68 |
| 3-aminoisobutyric acid | 6775 | 2099 | 16555 | 1982 | 0.41 | 0.67 | 2.30 |
| citramalic acid | 143 | 20 | 288 | 64 | 0.50 | 0.67 | 2.06 |
| tocopherol-beta | 612 | 136 | 9471 | 7592 | 0.06 | 0.67 | 2.04 |
| 4-hydroxymandelic acid | 187 | 28 | 335 | 70 | 0.56 | 0.67 | 1.92 |
| salicylaldehyde | 496 | 105 | 1291 | 363 | 0.38 | 0.67 | 1.91 |
| 3-hydroxyanthranilic acid | 89 | 16 | 172 | 38 | 0.52 | 0.67 | 1.91 |
| alpha-ketoglutarate | 504 | 45 | 856 | 160 | 0.59 | 0.67 | 1.90 |
| tocopherol gamma- | 502 | 91 | 8742 | 7688 | 0.06 | 0.67 | 1.83 |
| benzoic acid | 23520 | 4633 | 58996 | 17482 | 0.40 | 0.67 | 1.79 |
| **Jejunum** |  |  |  |  |  |  |  |
| cytidine | 1303 | 228 | 3027 | 641 | 0.43 | 0.43 | 2.65 |
| pseudo-uridine | 4244 | 421 | 7090 | 847 | 0.60 | 0.43 | 2.54 |
| 2-hydroxybutanoic acid | 12604 | 1684 | 6663 | 994 | 1.89 | 0.43 | 2.44 |
| alanine-alanine | 34870 | 2866 | 46016 | 1939 | 0.76 | 0.43 | 2.37 |
| lysine | 856542 | 114026 | 1218877 | 83670 | 0.70 | 0.43 | 2.29 |
| 3-hydroxypropionic acid | 42062 | 12269 | 10727 | 1841 | 3.92 | 0.43 | 2.24 |
| spermidine | 2264 | 373 | 3905 | 666 | 0.58 | 0.43 | 2.19 |
| 3-phenyllactic acid | 25235 | 6580 | 9195 | 3103 | 2.74 | 0.43 | 2.16 |
| 2'-deoxyguanosine | 4983 | 1124 | 12002 | 3231 | 0.42 | 0.43 | 2.16 |
| tyrosine | 1445802 | 134099 | 1809558 | 75879 | 0.80 | 0.43 | 2.14 |
| glycerol | 662917 | 65353 | 903704 | 67069 | 0.73 | 0.43 | 2.12 |
| succinic acid | 116068 | 18896 | 59680 | 8862 | 1.94 | 0.43 | 2.11 |
| 3,4-dihydroxycinnamic acid | 4848 | 1187 | 2141 | 447 | 2.26 | 0.43 | 2.09 |
| propane-1,3-diol | 22460 | 3907 | 11526 | 2229 | 1.95 | 0.47 | 2.02 |
| erythritol | 23142 | 5718 | 8321 | 1428 | 2.78 | 0.47 | 2.00 |
| D-erythro-sphingosine | 11283 | 2169 | 18853 | 2857 | 0.60 | 0.47 | 2.00 |
| 2-hydroxyglutaric acid | 7856 | 2520 | 2078 | 307 | 3.78 | 0.54 | 1.92 |
| indole-3-propionic acid | 644 | 70 | 2014 | 924 | 0.32 | 0.54 | 1.91 |
| taurine | 64332 | 17936 | 21855 | 6487 | 2.94 | 0.56 | 1.87 |
| glutamine | 426906 | 53406 | 529295 | 33975 | 0.81 | 0.56 | 1.86 |
| N-acetylglutamate | 5465 | 1555 | 2287 | 571 | 2.39 | 0.57 | 1.83 |
| dehydroascorbic acid | 7331 | 1286 | 23895 | 9299 | 0.31 | 0.57 | 1.82 |
| uridine | 4313 | 996 | 6222 | 1216 | 0.69 | 0.57 | 1.79 |
| histidine | 241424 | 33923 | 302012 | 18097 | 0.80 | 0.57 | 1.78 |
| **Ileum** |  |  |  |  |  |  |  |
| ribose | 136542 | 18599 | 69873 | 9895 | 1.95 | 0.23 | 2.64 |
| ornithine | 154202 | 17596 | 90865 | 12138 | 1.70 | 0.23 | 2.49 |
| 1,2-cyclohexanedione | 40090 | 5252 | 22087 | 3122 | 1.82 | 0.23 | 2.49 |
| oxoproline | 696873 | 35456 | 557521 | 27859 | 1.25 | 0.23 | 2.48 |
| nicotinic acid | 32179 | 2352 | 22147 | 3393 | 1.45 | 0.37 | 2.17 |
| glutamic acid | 764006 | 46051 | 578072 | 53726 | 1.32 | 0.37 | 2.16 |
| guanine | 8403 | 2229 | 4040 | 874 | 2.08 | 0.37 | 2.07 |
| citric acid | 8454 | 1425 | 15149 | 2289 | 0.56 | 0.37 | 2.04 |
| aspartic acid | 489606 | 31051 | 367615 | 43906 | 1.33 | 0.37 | 2.03 |
| inosine | 3733 | 1480 | 9082 | 2148 | 0.41 | 0.37 | 2.02 |
| 4-hydroxyphenylacetic acid | 3540 | 1032 | 1559 | 197 | 2.27 | 0.37 | 2.02 |
| cytidine-5-monophosphate | 664 | 81 | 1133 | 186 | 0.59 | 0.37 | 2.01 |
| N-acetylaspartic acid | 7455 | 1215 | 4111 | 721 | 1.81 | 0.37 | 2.00 |
| p-tolyl glucuronide | 552 | 88 | 930 | 162 | 0.59 | 0.37 | 2.00 |
| N-acetylputrescine | 7563 | 1419 | 3905 | 765 | 1.94 | 0.37 | 2.00 |
| adenine | 21073 | 6525 | 5897 | 1819 | 3.57 | 0.37 | 1.97 |
| 3-aminoisobutyric acid | 19066 | 1218 | 12956 | 2082 | 1.47 | 0.37 | 1.96 |
| urea | 60558 | 15440 | 127039 | 23784 | 0.48 | 0.37 | 1.95 |
| 3-4-hydroxyphenylpropionic acid | 3351 | 817 | 1803 | 276 | 1.86 | 0.45 | 1.84 |
| N-acetylglutamate | 6974 | 1757 | 3137 | 701 | 2.22 | 0.45 | 1.82 |
| UDP-glucuronic acid | 8523 | 1662 | 12428 | 1405 | 0.69 | 0.45 | 1.81 |
| sorbitol | 21692 | 3997 | 39473 | 6158 | 0.55 | 0.45 | 1.80 |
| thymine | 12806 | 1976 | 7944 | 1383 | 1.61 | 0.45 | 1.79 |
| N-acetylglycine | 17251 | 4355 | 8065 | 1831 | 2.14 | 0.45 | 1.79 |
| lactitol | 5533 | 2928 | 14641 | 5677 | 0.38 | 0.51 | 1.72 |
| glucose-6-phosphate | 758 | 303 | 1931 | 760 | 0.39 | 0.51 | 1.72 |

^1^Mean of normalized (mTIC) peak intensities (mz/rt) for human milk (HM) or milk formula (MF) after MetaboAnalyst analyses; n = 9 -15 per group

^2^SEM = Standard error of the mean

^3^Fold change of HM mean to MF mean

^4^FDR = Benjamini-Hochberg adjusted P-Value

^5^VIP = variable importance in projection in PLS-DA models using all annotated metabolites to compare HM and MF within each region.
