# Supplementary material for: Human Milk-Fed Piglets Have a Distinct Small Intestine and Circulatory Metabolome Profile Relative to That of Milk Formula-Fed Piglets
Source: mSystems. 2021 Feb 9;6(1):e01376-20. doi: 10.1128/mSystems.01376-20 (PMC7883546; doi:10.1128/mSystems.01376-20)
Supplement: TABLE S3 [file mSystems.01376-20-st003.docx]

| **Compound** | **HM^1^** | **SEM^2^** | **MF^1^** | **SEM^2^** | **FC^3^** | **FDR^5^** | **VIP^6^** |
| --- | --- | --- | --- | --- | --- | --- | --- |
| hydrocinnamic acid | 7781 | 306 | 5948 | 358 | 1.31 | 0.19 | 2.45 |
| phthalic acid | 27940 | 6060 | 12269 | 1553 | 2.28 | 0.19 | 2.21 |
| maltotriose | 252 | 25 | 176 | 12 | 1.43 | 0.19 | 2.21 |
| asparagine | 10909 | 657 | 14657 | 988 | 0.74 | 0.19 | 2.17 |
| xanthosine | 203 | 14 | 151 | 9 | 1.34 | 0.19 | 2.16 |
| isothreonic acid | 17379 | 795 | 13819 | 880 | 1.26 | 0.19 | 2.10 |
| nicotinic acid | 853 | 75 | 644 | 34 | 1.33 | 0.19 | 2.09 |
| 3-hydroxy-3-methylglutaric acid | 356 | 24 | 274 | 14 | 1.30 | 0.19 | 2.09 |
| guanine | 4143 | 335 | 2767 | 379 | 1.50 | 0.19 | 2.08 |
| UDP-N-acetylglucosamine | 326 | 28 | 221 | 22 | 1.47 | 0.19 | 2.06 |
| phenylacetic acid | 4603 | 431 | 3434 | 219 | 1.34 | 0.22 | 2.01 |
| uric acid | 122 | 15 | 79 | 7 | 1.54 | 0.23 | 1.97 |
| 3-hydroxypropionic acid | 19436 | 2382 | 12143 | 1048 | 1.60 | 0.24 | 1.93 |
| pseudo-uridine | 10086 | 529 | 8198 | 450 | 1.23 | 0.24 | 1.90 |
| phosphoenolpyruvate | 1010 | 63 | 1269 | 84 | 0.80 | 0.24 | 1.87 |
| methionine | 1344 | 100 | 1071 | 49 | 1.25 | 0.24 | 1.86 |
| salicylaldehyde | 691 | 45 | 550 | 33 | 1.26 | 0.24 | 1.86 |
| ethanolamine | 7024 | 424 | 5440 | 530 | 1.29 | 0.24 | 1.85 |
| 2,3-dihydroxybutanoic acid | 1597 | 161 | 1033 | 114 | 1.55 | 0.24 | 1.85 |
| thymine | 2396 | 309 | 1398 | 171 | 1.71 | 0.25 | 1.82 |
| norvaline | 1548 | 93 | 1284 | 43 | 1.21 | 0.26 | 1.81 |
| O-phosphoserine | 253 | 27 | 182 | 16 | 1.39 | 0.41 | 1.65 |
| myristic acid | 10508 | 2103 | 6686 | 408 | 1.57 | 0.41 | 1.62 |
| oleamide | 812 | 70 | 640 | 46 | 1.27 | 0.41 | 1.62 |
| indole-3-propionic acid | 2008 | 183 | 1498 | 166 | 1.34 | 0.41 | 1.62 |
| hippuric acid | 9050 | 833 | 6306 | 542 | 1.44 | 0.41 | 1.61 |
| adenosine-5-monophosphate | 168 | 17 | 819 | 429 | 0.21 | 0.41 | 1.60 |
| 7-methylguanine | 231 | 10 | 196 | 12 | 1.18 | 0.41 | 1.59 |
| glycolic acid | 47507 | 5418 | 32561 | 3476 | 1.46 | 0.41 | 1.58 |
| hydroquinone | 5877 | 774 | 9118 | 1190 | 0.64 | 0.41 | 1.57 |
| cysteine | 29690 | 1970 | 24446 | 1604 | 1.21 | 0.41 | 1.56 |
| 3-hydroxybenzoic acid | 811 | 261 | 334 | 152 | 2.43 | 0.42 | 1.55 |
| xylitol | 12870 | 720 | 10840 | 761 | 1.19 | 0.43 | 1.53 |
| urocanic acid | 311 | 26 | 251 | 10 | 1.24 | 0.43 | 1.52 |
| 6-hydroxynicotinic acid | 910 | 67 | 728 | 61 | 1.25 | 0.43 | 1.51 |

^1^Mean of normalized (mTIC) peak intensities (mz/rt) for human milk (HM) or milk formula (MF) after MetaboAnalyst analyses; n=15/group

^2^SEM = Standard error of the mean

^3^Fold change of HM mean to MF mean

^4^FDR = Benjamini-Hochberg adjusted P-Value

^5^VIP = variable importance in projection in PLS-DA models using all annotated metabolites to compare HM and MF within each region.
