# Supplementary material for: Human Milk-Fed Piglets Have a Distinct Small Intestine and Circulatory Metabolome Profile Relative to That of Milk Formula-Fed Piglets
Source: mSystems. 2021 Feb 9;6(1):e01376-20. doi: 10.1128/mSystems.01376-20 (PMC7883546; doi:10.1128/mSystems.01376-20)
Supplement: TABLE S4 [file mSystems.01376-20-st004.docx]

| **Compound** | **HM^1^** | **SEM^2^** | **MF^1^** | **SEM^2^** | **FC^3^** | ***P*^4^** | **FDR^5^** | **VIP^6^** |
| --- | --- | --- | --- | --- | --- | --- | --- | --- |
| glyceric acid | 145497 | 20718 | 72226 | 6126 | 2.01 | < 0.01 | 0.08 | 3.15 |
| galactonic acid | 596148 | 70135 | 307452 | 39949 | 1.94 | < 0.01 | 0.18 | 2.85 |
| pinitol | 21446 | 3184 | 9947 | 2494 | 2.16 | < 0.01 | 0.18 | 2.74 |
| sorbitol | 1736910 | 393770 | 501582 | 127076 | 3.46 | < 0.01 | 0.18 | 2.73 |
| shikimic acid | 41127 | 3579 | 24807 | 5064 | 1.66 | < 0.01 | 0.23 | 2.59 |
| conduritol-beta-expoxide | 18051 | 5840 | 39408 | 7063 | 0.46 | < 0.01 | 0.23 | 2.57 |
| alanine-alanine | 26981 | 4237 | 51057 | 9975 | 0.53 | 0.01 | 0.32 | 2.44 |
| pantothenic acid | 107600 | 6942 | 72473 | 8408 | 1.48 | 0.01 | 0.32 | 2.38 |
| melibiose | 2134 | 321 | 1064 | 134 | 2.01 | 0.01 | 0.32 | 2.37 |
| ribonic acid | 29486 | 3692 | 18103 | 2631 | 1.63 | 0.02 | 0.44 | 2.25 |
| parabanic acid | 70019 | 6665 | 47615 | 5257 | 1.47 | 0.02 | 0.44 | 2.23 |
| 3-hydroxyphenylacetic acid | 5739 | 1041 | 3126 | 297 | 1.84 | 0.03 | 0.63 | 2.06 |
| tartaric acid | 7696 | 2633 | 12239 | 2074 | 0.63 | 0.03 | 0.63 | 2.06 |
| linoleic acid | 8510 | 2273 | 3284 | 720 | 2.59 | 0.03 | 0.66 | 2.02 |
| xanthurenic acid | 1029 | 141 | 694 | 93 | 1.48 | 0.04 | 0.67 | 1.99 |
| galactitol | 436656 | 98408 | 224033 | 42085 | 1.95 | 0.04 | 0.68 | 1.93 |
| raffinose | 7932 | 960 | 5381 | 1197 | 1.47 | 0.05 | 0.68 | 1.89 |
| mucic acid | 1808 | 161 | 1159 | 120 | 1.56 | 0.05 | 0.68 | 1.87 |
| 2,6-diaminopimelic acid | 1533 | 128 | 2755 | 565 | 0.56 | 0.05 | 0.68 | 1.87 |
| 4-pyridoxic acid | 17770 | 1435 | 12949 | 1476 | 1.37 | 0.05 | 0.68 | 1.87 |
| 3-hydroxybenzoic acid | 2733 | 792 | 14304 | 6869 | 0.19 | 0.05 | 0.68 | 1.86 |
| alanine | 271248 | 37269 | 494645 | 143531 | 0.55 | 0.05 | 0.70 | 1.83 |

^1^Mean of normalized (mTIC) peak intensities (mz/rt) for human milk (HM) or milk formula (MF) after MetaboAnalyst analyses; n = 15 per group

^2^SEM = Standard error of the mean

^3^Fold change of HM mean to MF mean

^4^P-Value ≤ 0.05

^5^FDR = Benjamini-Hochberg adjusted P-Value

^6^VIP = variable importance in projection in PLS-DA models using all annotated metabolites to compare HM and MF within each intestinal section.
