# Supplementary figures and images for: Human Milk-Fed Piglets Have a Distinct Small Intestine and Circulatory Metabolome Profile Relative to That of Milk Formula-Fed Piglets
Source: mSystems. 2021 Feb 9;6(1):e01376-20. doi: 10.1128/mSystems.01376-20 (PMC7883546; doi:10.1128/mSystems.01376-20)

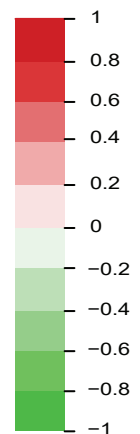

A.

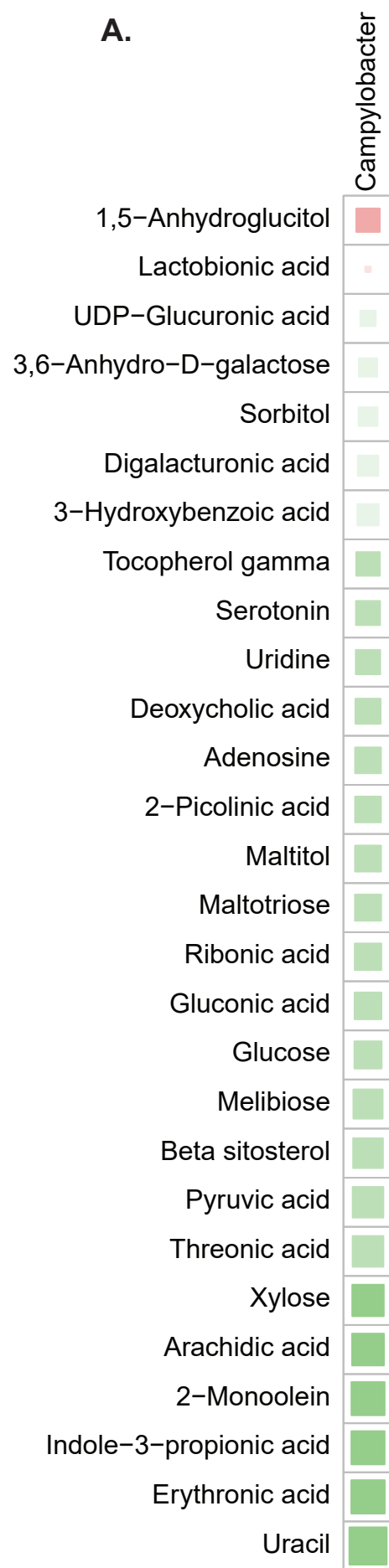

B.

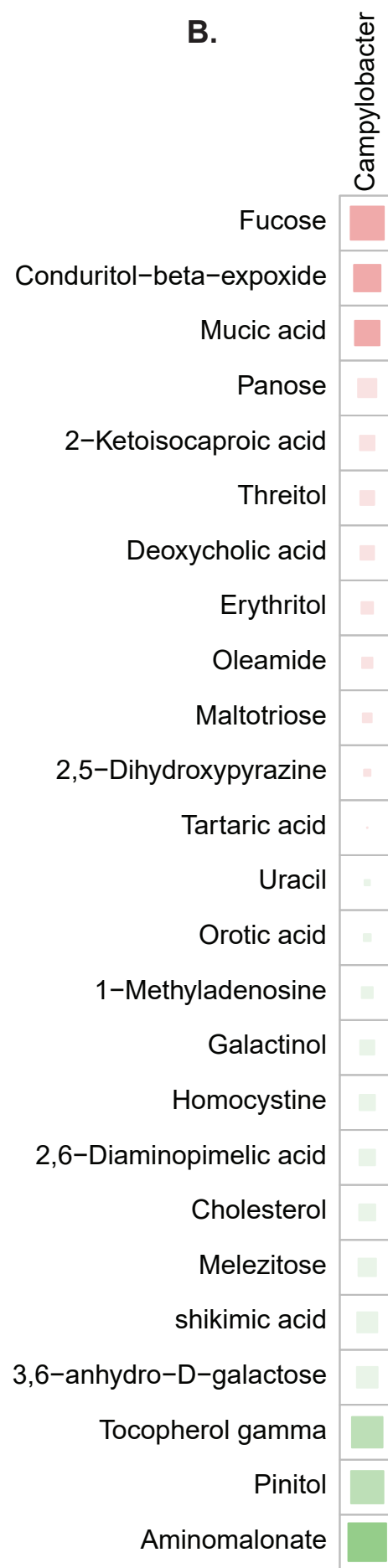

C.

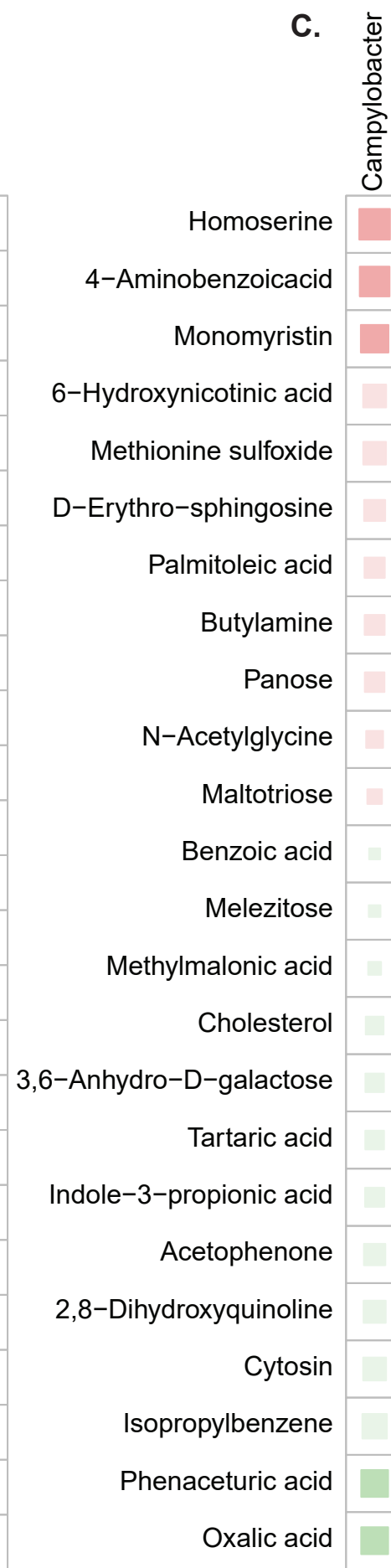

Clostridium

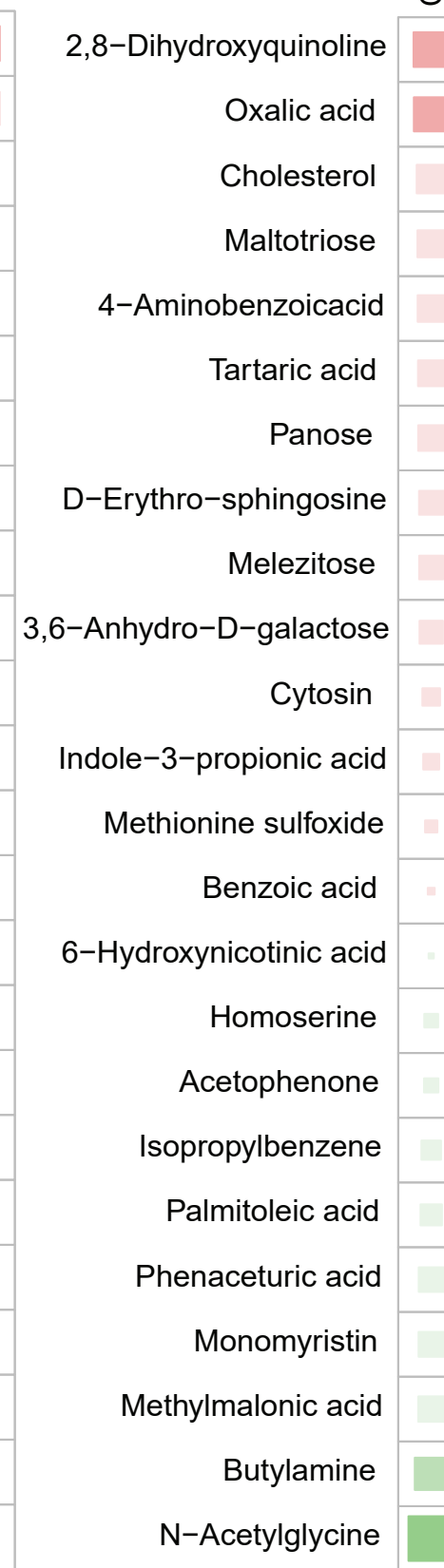

Turicibacter

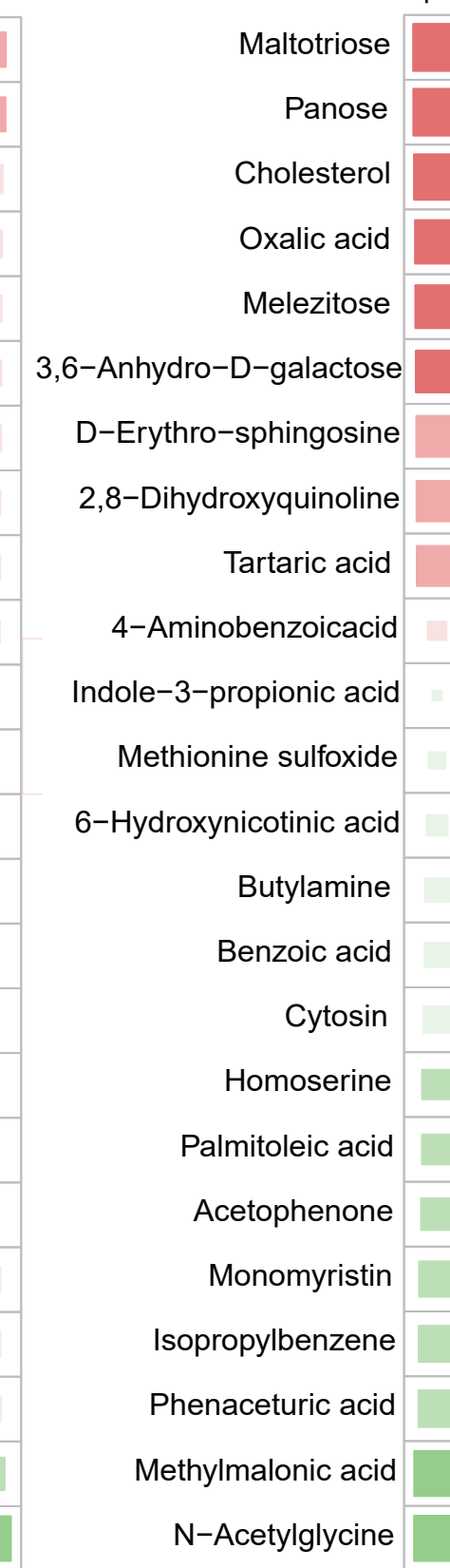

Veillonella

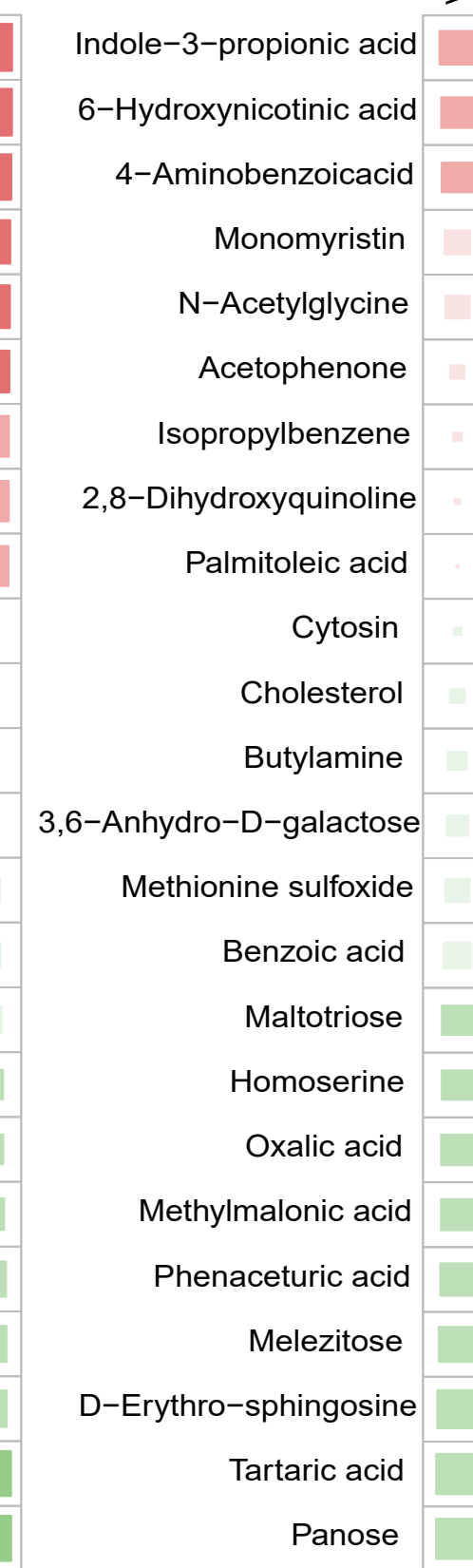

Supplement: FIG S1 [file mSystems.01376-20-sf001.pdf]

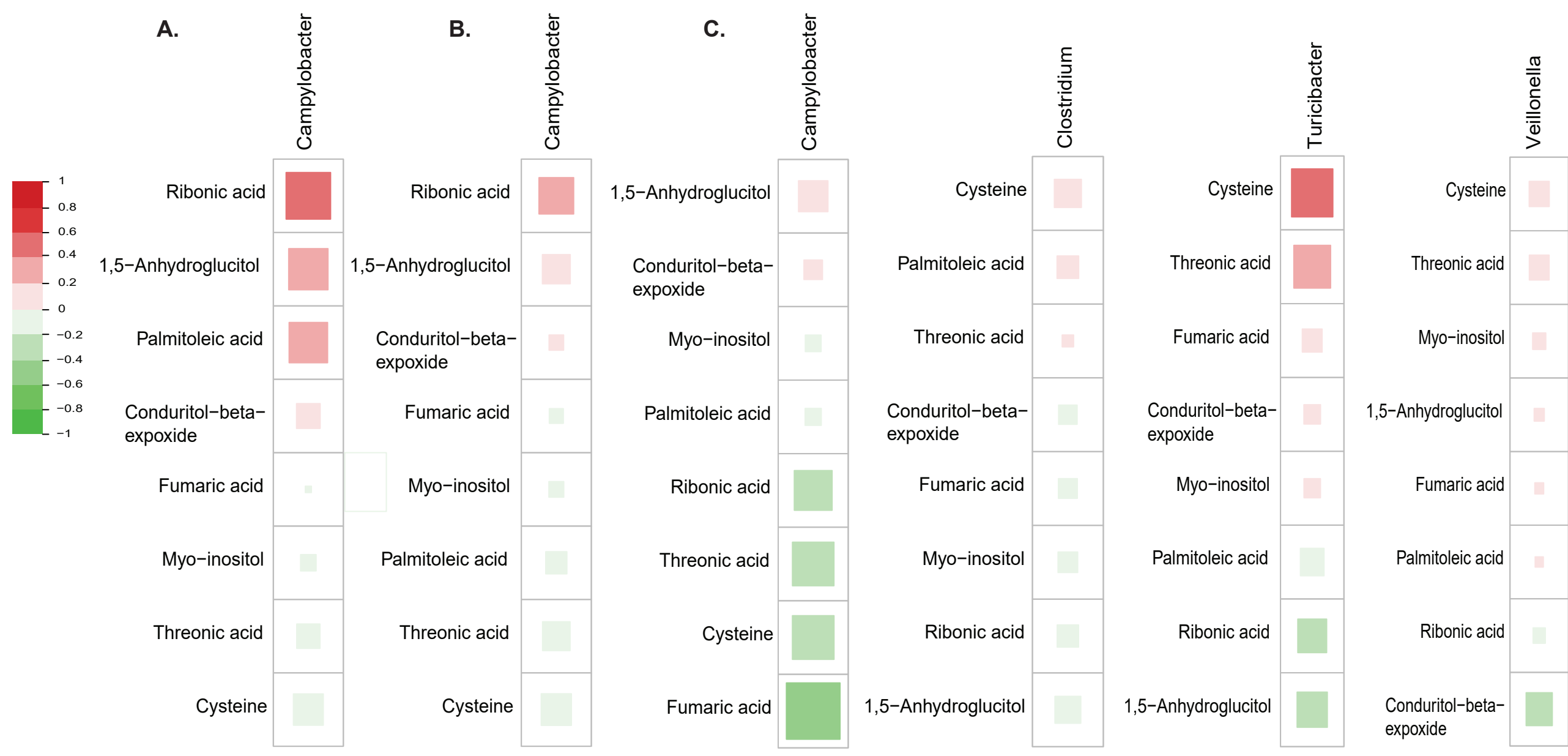

Supplement: FIG S2 [file mSystems.01376-20-sf002.pdf]

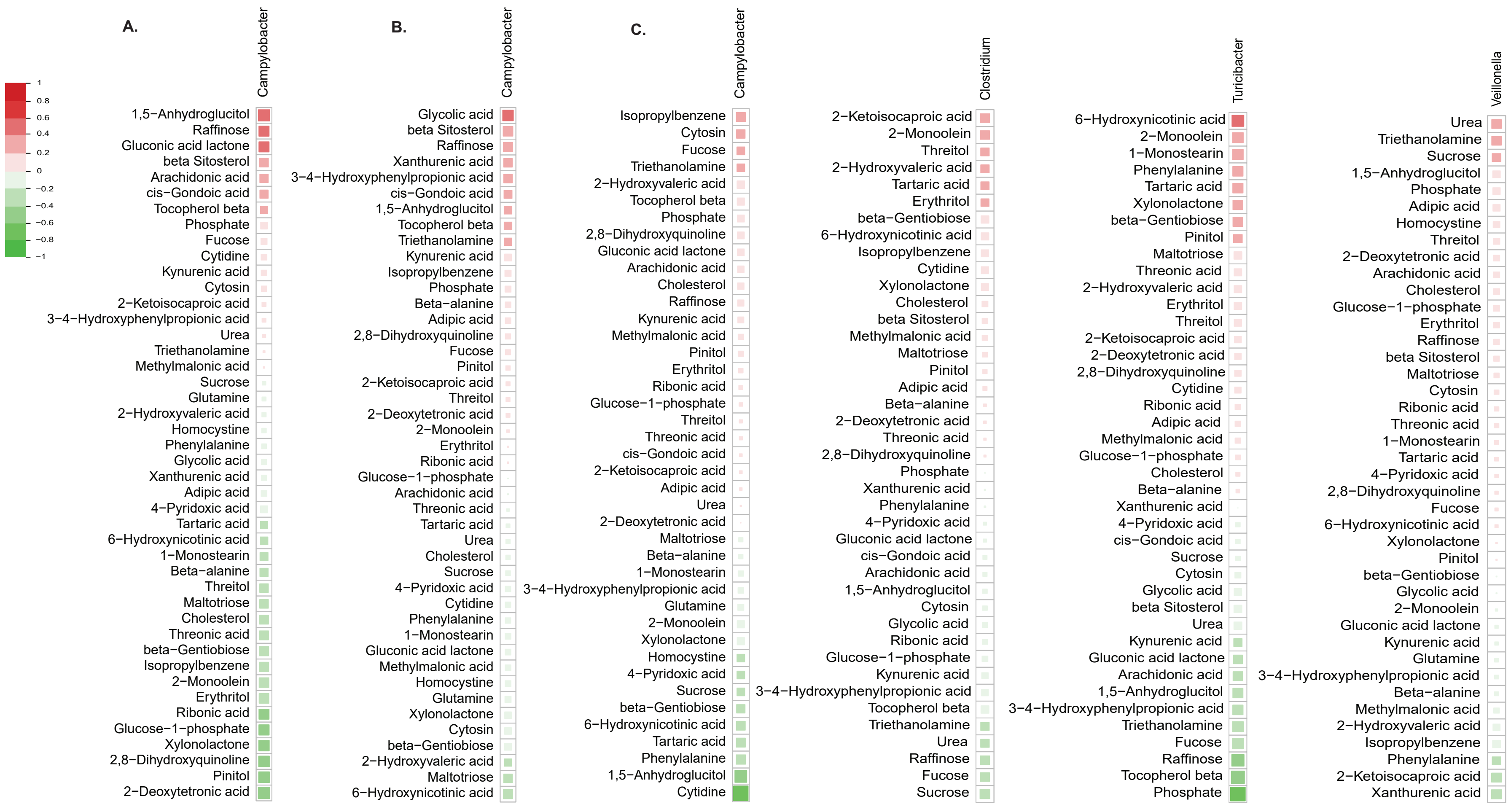

Supplement: FIG S3 [file mSystems.01376-20-sf003.pdf]

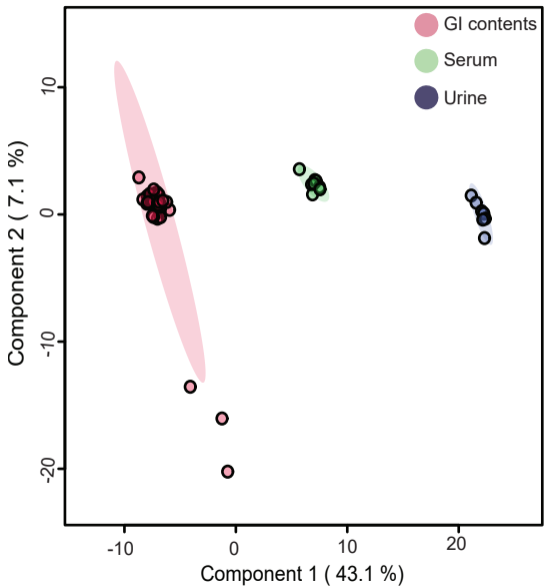

Supplement: FIG S4 [file mSystems.01376-20-sf004.pdf]
