# Supplementary material for: Human Milk-Fed Piglets Have a Distinct Small Intestine and Circulatory Metabolome Profile Relative to That of Milk Formula-Fed Piglets
Source: mSystems. 2021 Feb 9;6(1):e01376-20. doi: 10.1128/mSystems.01376-20 (PMC7883546; doi:10.1128/mSystems.01376-20)
Supplement: TABLE S5 [file mSystems.01376-20-st005.docx]

| **Serum** | **Degrees of Freedom** | **Sums of Sqs** | **F. Model** | ***P*r (> F)** |
| --- | --- | --- | --- | --- |
| Diet | 1 | 0.02 | 1.20 | 0.30 |
| immunization | 1 | 0.01 | 0.80 | 0.60 |
| Diet:immunization | 1 | 0.02 | 1.23 | 0.30 |
| Residuals | 25 | 0.42 |  |  |
| **Urine** | **Degrees of Freedom** | **Sums of Sqs** | **F. Model** | ***P*r (> F)** |
| Diet | 1 | 0.08 | 1.01 | 0.34 |
| immunization | 1 | 0.10 | 1.35 | 0.21 |
| Diet:immunization | 1 | 0.05 | 0.62 | 0.68 |
| Residuals | 23 | 1.81 |  |  |
| **Duodenum** | **Degrees of Freedom** | **Sums of Sqs** | **F. Model** | ***P*r (> F)** |
| Diet | 1 | 0.07 | 1.75 | 0.13 |
| immunization | 1 | 0.02 | 0.42 | 0.87 |
| Diet:immunization | 1 | 0.18 | 4.73 | 0.003 |
| Residuals | 12 | 0.47 |  |  |
| **Jejunum** | **Degrees of Freedom** | **Sums of Sqs** | **F. Model** | ***P*r (> F)** |
| Diet | 1 | 0.05 | 1.20 | 0.27 |
| immunization | 1 | 0.00 | 0.08 | 0.99 |
| Diet:immunization | 1 | 0.05 | 1.17 | 0.30 |
| Residuals | 26 | 1.07 |  |  |
| **Ileum** | **Degrees of Freedom** | **Sums of Sqs** | **F. Model** | ***P*r (> F)** |
| Diet | 1 | 0.07 | 1.60 | 0.16 |
| immunization | 1 | 0.13 | 3.07 | 0.03 |
| Diet:immunization | 1 | 0.14 | 3.28 | 0.01 |
| Residuals | 26 | 1.16 |  |  |
